# Personalized predictions of patient outcomes during and after hospitalization using artificial intelligence

**DOI:** 10.1038/s41746-020-0249-z

**Published:** 2020-04-03

**Authors:** C. Beau Hilton, Alex Milinovich, Christina Felix, Nirav Vakharia, Timothy Crone, Chris Donovan, Andrew Proctor, Aziz Nazha

**Affiliations:** 1 Center for Clinical Artificial Intelligence, Cleveland Clinic, Cleveland, OH 44121 USA; 2 Cleveland Clinic Lerner College of Medicine of Case Western Reserve University, Cleveland, OH 44121 USA; 3 Taussig Cancer Institute, Cleveland Clinic, Cleveland, OH 44121 USA; 4 Department of Quantitative Health Sciences, Cleveland Clinic, Cleveland, OH 44121 USA; 5 Department of Quantitive Health Sciences, Cleveland Clinic, Cleveland, OH 44121 USA; 6 Department of Internal Medicine, Cleveland Clinic Community Care, Cleveland Clinic, Cleveland, OH 44121 USA; 7 Enterprise Business Intelligence & Analytics, Cleveland Clinic, Cleveland, OH 44121 USA

**Keywords:** Risk factors, Health care economics, Outcomes research

## Abstract

Hospital systems, payers, and regulators have focused on reducing length of stay (LOS) and early readmission, with uncertain benefit. Interpretable machine learning (ML) may assist in transparently identifying the risk of important outcomes. We conducted a retrospective cohort study of hospitalizations at a tertiary academic medical center and its branches from January 2011 to May 2018. A consecutive sample of all hospitalizations in the study period were included. Algorithms were trained on medical, sociodemographic, and institutional variables to predict readmission, length of stay (LOS), and death within 48–72 h. Prediction performance was measured by area under the receiver operator characteristic curve (AUC), Brier score loss (BSL), which measures how well predicted probability matches observed probability, and other metrics. Interpretations were generated using multiple feature extraction algorithms. The study cohort included 1,485,880 hospitalizations for 708,089 unique patients (median age of 59 years, first and third quartiles (QI) [39, 73]; 55.6% female; 71% white). There were 211,022 30-day readmissions for an overall readmission rate of 14% (for patients ≥65 years: 16%). Median LOS, including observation and labor and delivery patients, was 2.94 days (QI [1.67, 5.34]), or, if these patients are excluded, 3.71 days (QI [2.15, 6.51]). Predictive performance was as follows: 30-day readmission (AUC 0.76/BSL 0.11); LOS > 5 days (AUC 0.84/BSL 0.15); death within 48–72 h (AUC 0.91/BSL 0.001). Explanatory diagrams showed factors that impacted each prediction.

## Introduction

Patients and providers face a great amount of uncertainty before, during, and after hospital encounters. Predictive modeling holds promise for identifying patients at the highest risk for adverse events, such as extended length of stay (LOS), 30-day readmission, and death within the hospital encounter. Despite the success of predictive models in achieving discriminatory power in these and other areas, simplistic models cannot account for complicated intersections of medical, institutional, and demographic factors. Conversely, complex models that account for these interactions are difficult or impossible to interpret or audit, and therefore may be inactionable or harmful if put into use, and can also be difficult for healthcare providers to understand or accept^1–3^. Recent studies suggest that a focus on metrics such as 30-day readmission without addressing underlying causes may lead to increased patient mortality and increased cost without improving patient outcomes^4^.

Significant recent advances in artificial intelligence (AI), machine learning (ML), and deep learning (DL) have yielded compelling innovations including self-driving cars^5^, product recommendations^6^, and superhuman performance in complex games, such as chess and Go^7^. These advances have also started to impact healthcare, particularly in visual tasks: detecting diabetic retinopathy in ophthalmology images^8^, cancers in biopsy slides^9^, and malignant versus benign skin lesions, often with accuracy comparable to or exceeding trained physicians^10^. As electronic healthcare record (EHR) data increase in size and complexity, AI and ML may provide predictive modeling tools that can improve patient safety and outcomes while decreasing cost. A major hurdle for ML in healthcare is the “black box phenomenon,” or lack of explainability, to patients and healthcare providers. However, recent advances have provided algorithms that reliably extract important variables and explain model decisions, allowing for auditing and exploration. Such approaches can ensure that variables included in the final model are clinically relevant and can be recognized and understood and may lead to new insights and hypotheses. Most importantly, explainable ML supports clinician and patient decision-making, rather than supplants it, by making explicit the natures and characters of the variables the algorithm considered most important when making its predictions.

In this study, we hypothesized that interpretable predictive models would achieve comparable or superior performance to existing models and enable an understanding of factors associated with adverse outcomes. Here we report ML models with high predictive power for readmission and extended LOS, along with patient-level and cohort-level interpretations, and discuss the use of ML as a tool to aid understanding.

## Results

### Study cohort

In the study period, there were 1,485,880 hospitalizations for 708,089 unique patients, 439,696 (62%) of whom had only 1 hospitalization recorded. The median number of hospitalizations per patient was 1 (first and third quartile (QI) [1.0, 2.0]). There were 211,022 30-day readmissions for an overall readmission rate of 14%. Among patients aged ≥65 years, the 30-day readmission rate was 16%. The median LOS, including patients in observation status and labor and delivery patients, was 2.94 days (QI [1.67, 5.34]), or if these patients are excluded, 3.71 days (QI [2.15, 6.51]). The demographic and clinical characteristics of the patient cohort are summarized in Table 1. Higher rates of 30-day readmissions were observed in patients who were older (median age 62 vs. 59 years), African American (rate of 17% vs. 13% in whites), divorced/separated or widowed (17% vs. 13% in married/partnered or single patients), on Medicare insurance (rate of 17% vs. 10% for private insurance), and had one or multiple chronic conditions such as cancer, renal disease, congestive heart failure, chronic obstructive pulmonary disease, etc. (Table 1).

**Table 1:** Characteristics of hospital encounters in the study sample, overall and according to readmission and extended length of stay.

Characteristic	Overall	Not readmitted within 30 days	Readmitted within 30 days	Hospital stay less than 5 days	Hospital stay over 5 days
Number of hospitalizations	1,485,880	1,274,858	211,022	1,234,148	251,732
Age, median [Q1, Q3]	59.0 [39.0, 73.0]	59.0 [38.0, 73.0]	62.0 [48.0, 76.0]	58.0 [36.0, 72.0]	66.0 [54.0, 78.0]
Female, *n* (%)	826,025 (55.6)	713,391 (56.0)	112,634 (53.4)	698,382 (56.6)	127,643 (50.7)
Race/ethnicity, *n* (%)					
African American	333,212 (22.4)	276,208 (21.7)	57,004 (27.0)	276,476 (22.4)	56,736 (22.5)
White	1,055,180 (71.1)	913,085 (71.7)	142,095 (67.4)	873,453 (70.8)	181,727 (72.2)
Other	96,592 (6.5)	84,755 (6.7)	11,837 (5.6)	83,453 (6.8)	13,139 (5.2)
Marital status, *n* (%)					
Divorced or separated	134,841 (9.1)	111,680 (8.8)	23,161 (11.0)	108,779 (8.8)	26,062 (10.4)
Married or partnered	594,375 (40.0)	515,620 (40.5)	78,755 (37.3)	494,338 (40.1)	100,037 (39.7)
Single	554,116 (37.3)	477,592 (37.5)	76,524 (36.3)	472,301 (38.3)	81,815 (32.5)
Widowed	175,822 (11.8)	146,611 (11.5)	29,211 (13.8)	136,888 (11.1)	38,934 (15.5)
Other	26,200 (1.8)	22,847 (1.8)	3353 (1.6)	21,347 (1.7)	4853 (1.9)
Payer class, *n* (%)					
Medicaid	221,969 (16.4)	188,630 (16.3)	33,339 (17.0)	193,978 (17.2)	27,991 (12.1)
Medicare	725,125 (53.5)	601,752 (51.9)	123,373 (63.0)	567,435 (50.5)	157,690 (68.4)
Private health insurance	329,842 (24.3)	298,444 (25.7)	31,398 (16.0)	293,292 (26.1)	36,550 (15.9)
Other	78,269 (5.8)	70,553 (6.1)	7716 (3.9)	69,940 (6.2)	8329 (3.6)
Comorbidities, *n* (%)					
Cancer	183,367 (12.3),	142,205 (11.2)	41,162 (19.5)	140,188 (11.4)	43,179 (17.2)
Metastatic solid tumor	55,906 (3.8)	41,867 (3.3)	14,039 (6.7)	42,339 (3.4)	13,567 (5.4)
Solid organ transplant	33,780 (2.3)	24,928 (2.0)	8852 (4.2)	22,837 (1.9)	10,943 (4.3)
AIDS/HIV	4552 (0.3)	3310 (0.3)	1242 (0.6)	3703 (0.3)	849 (0.3)
Renal disease	177,544 (11.9)	133,099 (10.4)	44,445 (21.1)	129,114 (10.5)	48,430 (19.2)
Mild liver disease	93,947 (6.3)	71,396 (5.6)	22,551 (10.7)	73,362 (5.9)	20,585 (8.2)
Moderate or severe liver disease	22,816 (1.5)	15,542 (1.2)	7274 (3.4)	15,971 (1.3)	6845 (2.7)
Diabetes with chronic complication	125,118 (8.4)	95,619 (7.5)	29,499 (14.0)	95,561 (7.7)	29,557 (11.7)
Diabetes without chronic complication	293,379 (19.7)	232,187 (18.2)	61,192 (29.0)	226,901 (18.4)	66,478 (26.4)
Hypertension	939,048 (63.2)	779,460 (61.1)	159,588 (75.6)	744,603 (60.3)	194,445 (77.2)
Myocardial infarction	69,914 (4.7)	53,267 (4.2)	16,647 (7.9)	52,835 (4.3)	17,079 (6.8)
Congestive heart failure	215,510 (14.5)	164,879 (12.9)	50,631 (24.0)	155,898 (12.6)	59,612 (23.7)
Cerebrovascular disease	193,243 (13.0)	154,368 (12.1)	38,875 (18.4)	148,158 (12.0)	45,085 (17.9)
Chronic obstructive pulmonary disease	302,548 (20.4)	240,195 (18.8)	62,353 (29.5)	238,907 (19.4)	63,641 (25.3)
Pneumonia	188,684 (12.7)	142,066 (11.1)	46,618 (22.1)	142,437 (11.5)	46,247 (18.4)
Dementia	56,876 (3.8)	45,461 (3.6)	11,415 (5.4)	41,554 (3.4)	15,322 (6.1)
Anxiety	181,440 (12.2)	146,263 (11.5)	35,177 (16.7)	150,668 (12.2)	30,772 (12.2)
Depression	259,323 (17.5)	207,914 (16.3)	51,409 (24.4)	212,806 (17.2)	46,517 (18.5)
Psychosis	52,085 (3.5)	39,086 (3.1)	12,999 (6.2)	38,544 (3.1)	13,541 (5.4)
Receiving dialysis	17,791 (1.2)	12,604 (1.0)	5187 (2.5)	10,658 (0.9)	7133 (2.8)
Selected discharge laboratory results, *n* (%)					
Low hemoglobin level (<12 g/dL)	248,387 (16.7)	204,139 (16.0)	44,248 (21.0)	200,374 (16.2)	48,013 (19.1)
Low sodium level (<135 mEq/L)	38,847 (2.6)	31,439 (2.5)	7408 (3.5)	29,467 (2.4)	9380 (3.7)
Hospital encounter information, median [Q1, Q3] or *n* (%)					
Previous hospitalizations	1.0 [0.0, 2.0]	0.0 [0.0, 2.0]	2.0 [0.0, 6.0]	1.0 [0.0, 2.0]	1.0 [0.0, 3.0]
Emergency department (ED) admission	725,843 (48.8)	603,317 (47.3)	122,526 (58.1)	618,055 (50.1)	107,788 (42.8)
Any ED visits in the past 6 months	644,102 (43.3)	511,323 (40.1)	132,779 (62.9)	521,248 (42.2)	122,854 (48.8)
Total ED visits in the past 6 months	0.0 [0.0, 1.0]	0.0 [0.0, 1.0]	1.0 [0.0, 3.0]	0.0 [0.0, 1.0]	0.0 [0.0, 2.0]
Admission class, *n* (%)					
Ambulatory surgical procedures	8081 (0.5)	7464 (0.6)	617 (0.3)	8060 (0.7)	21 (0.0)
Emergency	7058 (0.5)	6417 (0.5)	641 (0.3)	7055 (0.6)	3 (0.0)
Hospice	1486 (0.1)	1463 (0.1)	23 (0.0)	1357 (0.1)	129 (0.1)
Inpatient	1,185,985 (80.0)	1,011,772 (79.6)	174,213 (82.7)	937,614 (76.2)	248,371 (98.7)
Observation	261,942 (17.7)	228,559 (18.0)	33,383 (15.8)	260,955 (21.2)	987 (0.4)
Outpatient	10,559 (0.7)	9415 (0.7)	1144 (0.5)	10,513 (0.9)	46 (0.0)
Psychiatric inpatient	3381 (0.2)	2936 (0.2)	445 (0.2)	2198 (0.2)	1183 (0.5)
Other	4074 (0.3)	3799 (0.3)	275 (0.1)	3082 (0.3)	992 (0.4)
Discharge location, *n* (%)					
Expired	18,615 (1.4)	18,615 (1.6)	0 (0.0)	10,907 (1.0)	7708 (3.3)
General acute care hospital	19,855 (1.5)	17,490 (1.5)	2365 (1.2)	16,105 (1.4)	3750 (1.6)
Home	959,559 (71.1)	833,797 (72.2)	125,762 (64.8)	862,810 (77.1)	96,749 (42.0)
Home care services	134,970 (10.0)	109,327 (9.5)	25,643 (13.2)	93,833 (8.4)	41,137 (17.9)
Hospice	14,318 (1.1)	13,765 (1.2)	553 (0.3)	8879 (0.8)	5439 (2.4)
Intermediate care facility	9046 (0.7)	7451 (0.6)	1595 (0.8)	5215 (0.5)	3831 (1.7)
Left against medical advice	13,864 (1.0)	10,599 (0.9)	3265 (1.7)	13,374 (1.2)	490 (0.2)
Long-term care facility	14,592 (1.1)	12,210 (1.1)	2382 (1.2)	5403 (0.5)	9189 (4.0)
Skilled nursing facility	145,882 (10.8)	115,106 (10.0)	30,776 (15.8)	87,530 (7.8)	58,352 (25.3)
Transfer to a psychiatric hospital	6828 (0.5)	6276 (0.5)	552 (0.3)	6197 (0.6)	631 (0.3)
Transfer to another hospital	4482 (0.3)	4032 (0.3)	450 (0.2)	3797 (0.3)	685 (0.3)
Other	7109 (0.5)	6240 (0.5)	869 (0.4)	4740 (0.4)	2369 (1.0)
Outcomes of interest					
30-day readmissions, *n* (%)	211,022 (14.2)	0 (0.0)	211,022 (100.0)	158,577 (12.8)	52,445 (20.8)
Length of stay in days, median [Q1, Q3]	2.9 [1.7, 5.3]	2.8 [1.6, 5.1]	3.9 [2.0, 7.0]	2.4 [1.4, 3.9]	10.6 [8.3, 15.0]

### Prediction of inpatient outcomes

**Fig. 1:**
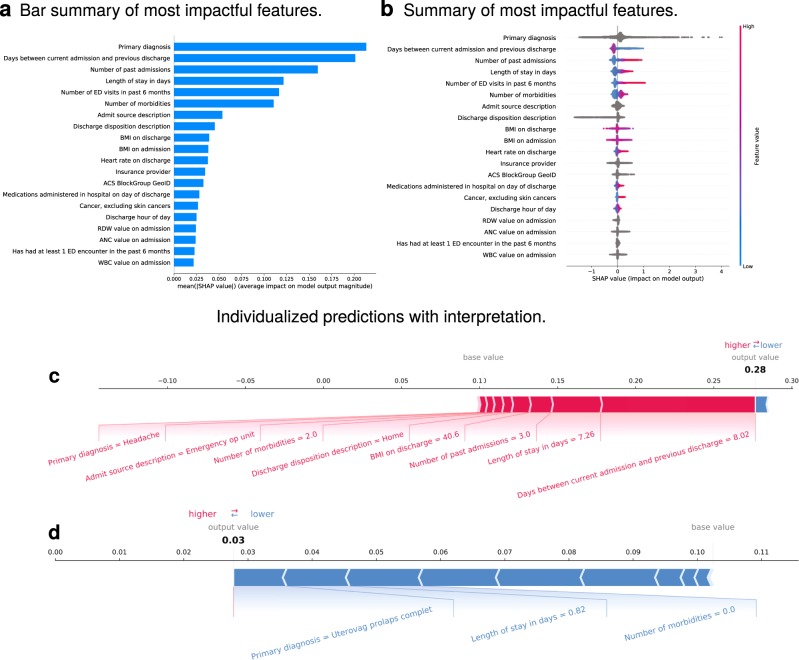
30-Day readmission. **a** Shows the most impactful features on prediction (ranked from most to least important). **b** Shows the distribution of the impacts of each feature on the model output. The colors represent the feature values for numeric features: red for larger values and blue for smaller. The line is made of individual dots representing each admission, and the thickness of the line is determined by the number of examples at a given value (for example, most patients have a low number of past admissions). A negative SHAP value (extending to the left) indicates a reduced probability, while a positive one (extending to the right) indicates an increased probability. For non-numeric features, such as primary diagnosis, the gray points represent specific possible values, with certain diagnoses greatly increasing or reducing the model’s output, while the majority of diagnoses have relatively mild impact on prediction. **c**, **d** Show the composition of individualized predictions for two patients. The patient in **c** was admitted from the emergency outpatient unit with a headache and stayed for >7 days. In addition, this patient had been hospitalized 3 times prior to this admission and had been discharged from the last admission only 8 days prior. The predicted probability of 30-day readmission (~0.30) was three times the baseline value predicted by the model (~0.1). All of the listed features increased the model’s prediction of risk by the relative amounts shown by the size of the red bars. Conversely, the patient in **d** was admitted for a complete uterovaginal prolapse, stayed less than a full day, and had no reported comorbidities, such as hypertension, depression, or a history of cancer. The model predicted their probability of 30-day readmission at 0.03 or roughly one-third of the baseline prediction. The top variables that contribute and will fit on the chart are shown, but the others can be queried in the live system. The model considers all variables, and SHAP reports on all variables internally, but the images are understandably truncated for visibility.

Thirty-day readmissions were predicted with an area under the receiver operator characteristic curve (ROC AUC, here abbreviated as simply “AUC”) of 0.76 (Supplementary Fig. 1a). The Brier score loss (BSL) was 0.11, calibration curve shown in Supplementary Fig. 1b. Average precision was 0.38 (see Supplementary Fig. 2c). Other off-the-shelf ML models, including a deep neural network, were trained on the same task, with performance generally inferior to the Gradient Boosting Machine (GBM), or in the case of the deep neural network, similar (see Supplementary Fig. 2 and Supplementary Table 1). When trained and evaluated on a smaller cohort of 300,000 hospitalizations, performance metrics were similar: AUC 0.75, BSL 0.11. The most impactful features included (ranked from the most to the least important): primary diagnosis, days between the current admission and the previous discharge, number of past admissions, LOS, total emergency department visits in the past 6 months, number of reported comorbidities, admission source, discharge disposition, and Body Mass Index (BMI) on admission and discharge, as well as others (Fig. 1a, b, see also Supplementary Fig. 3). Including more than the top ten variables in the model did not improve predictive power for the cohort overall but does allow for more specific rationale for prediction for certain patients, as well as examination of feature interactions for further exploration. Sample individualized predictions with their explanations are shown in Fig. 1c, d, and further examples are shown in Supplementary Fig. 4. The examples in Supplementary Fig. 4 show patients with comparable predicted probabilities but different compositions of features leading to these predictions.

In order to examine possible changes in causes of readmission risk as a function of time from discharge, we predicted readmission risk for several readmission thresholds and calculated SHAP (SHapley Additive exPlanation) for each. SHAP values for 3- and 7-day readmission are shown in Supplementary Fig. 5a, b, respectively. For example, 7-day readmission risk prediction achieved AUC of 0.70 with a BSL of 0.05 (Table 2). The most impactful feature remained primary diagnosis, but other features played more important roles—e.g., BlockGroup rose to second most important variable (from ninth), number of emergency department visits in the past 6 months rose to third importance from fourth, admission blood counts increased in importance, and insurance provider rose to eighth from twelfth. BMI on admission fell several places, and BMI on discharge no longer features in the top variables. The BMI variables are unique in that missing values tend to be important, in addition to extreme values, perhaps correlating with disease burden and/or hospital practices that could be further investigated.

**Table 2:** Performance of predictive models.

Target	ROC AUC	Average precision	Precision	Recall	Accuracy	F1 Score	Matthews correlation coefficient	Brier score loss	RMSE
Readmitted within 30 days	0.758 [0.755 to 0.762]	0.383 [0.377 to 0.388]	0.632 [0.620 to 0.647]	0.102 [0.098 to 0.106]	0.861 [0.860 to 0.861]	0.176 [0.169 to 0.182]	0.214 [0.208 to 0.220]	0.108 [0.108 to 0.109]	—
Readmitted within 7 days	0.701 [0.696 to 0.707]	0.127 [0.122 to 0.133]	0.586 [0.455 to 0.722]	0.003 [0.002 to 0.004]	0.949 [0.949 to 0.949]	0.006 [0.004 to 0.008]	0.040 [0.030 to 0.051]	0.047 [0.047 to 0.047]	—
Readmitted within 5 days	0.691 [0.684 to 0.698]	0.091 [0.086 to 0.095]	0.456 [0.000 to 1.000]	0.000 [0.000 to 0.001]	0.963 [0.963 to 0.963]	0.001 [0.000 to 0.002]	0.013 [−0.001 to 0.029]	0.035 [0.035 to 0.035]	—
Readmitted within 3 days	0.681 [0.674 to 0.689]	0.057 [0.053 to 0.062]	0.000 [0.000 to 0.000]	0.000 [0.000 to 0.000]	0.978 [0.978 to 0.978]	0.000 [0.000 to 0.000]	0.000 [0.000 to 0.000]	0.021 [0.021 to 0.021]	—
Days to readmission^a^	—	—	—	—	—	—	—	—	8.98
Death within 48–72 h^a^	0.91	—	—	—	—	—	—	0.001	—
Hospital stay >7 days	0.830 [0.827 to 0.833]	0.567 [0.561 to 0.572]	0.653 [0.646 to 0.659]	0.331 [0.325 to 0.337]	0.827 [0.825 to 0.828]	0.439 [0.434 to 0.445]	0.378 [0.371 to 0.384]	0.122 [0.121 to 0.123]	—
Hospital stay >5 days	0.829 [0.827 to 0.832]	0.705 [0.701 to 0.710]	0.690 [0.685 to 0.695]	0.546 [0.541 to 0.552]	0.767 [0.765 to 0.770]	0.609 [0.605 to 0.614]	0.453 [0.447 to 0.459]	0.155 [0.154 to 0.157]	—
Hospital stay >3 days	0.824 [0.822 to 0.827]	0.861 [0.859 to 0.864]	0.760 [0.758 to 0.762]	0.842 [0.839 to 0.845]	0.752 [0.749 to 0.754]	0.799 [0.797 to 0.801]	0.480 [0.475 to 0.485]	0.166 [0.165 to 0.167]	—
Length of stay (days)^a^	—	—	—	—	—	—	—	—	3.94

^a^Performance on these predictive tasks was poor to the extent that rigorous cross-validation was not performed.

**Fig. 2:**
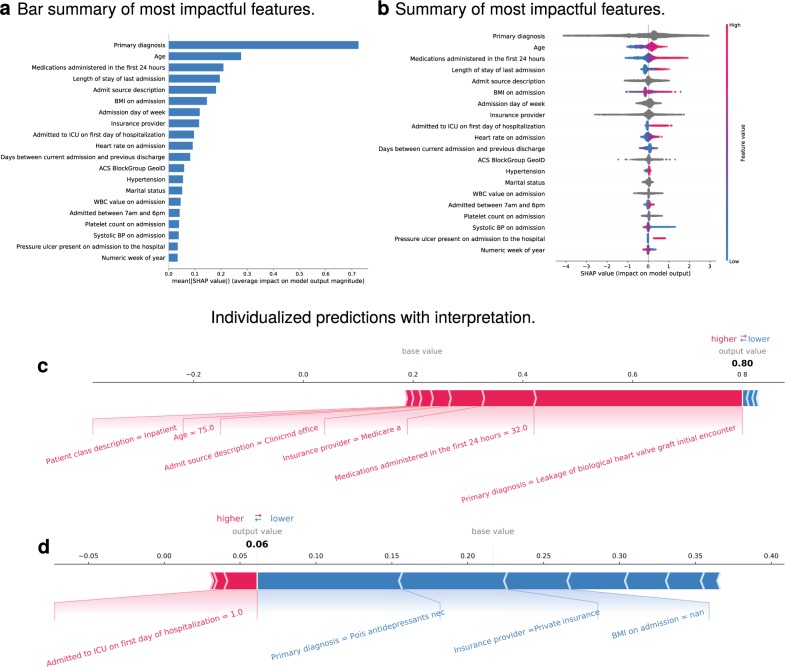
Length of stay >5 days. **a** shows the most impactful features on prediction (ranked from most to least important). **b** shows the distribution of the impacts of each feature on the model output. The colors represent the feature values for numeric features: red for larger values and blue for smaller. The line is made of individual dots representing each admission, and the thickness of the line is determined by the number of examples at a given value (for example, many of our patients are elderly). A negative SHAP value (extending to the left) indicates a reduced probability, while a positive one (extending to the right) indicates an increased probability. For example, advanced age increases the probability of extended length of stay (SHAP value between zero and one), while young age tends toward a SHAP value between roughly −1 and zero, corresponding to reduced probability. For non-numeric features, such as primary diagnosis, the gray points represent specific possible values, with certain diagnoses greatly increasing or reducing the model’s output, while the majority of diagnoses have relatively mild impact on prediction. **c**, **d** show the composition of individualized predictions for two patients. The 75-year-old patient in **c** was admitted to the inpatient service directly from a physician’s office with leakage of a heart valve graft. The patient received 32 medications in the first 24 h and has Medicare Part A insurance coverage. The model predicted that the patient’s probability of staying >5 days was 0.80, nearly four times the baseline prediction of ~0.2. The majority of the model’s prediction was based on the diagnosis, followed by the number of initial medications, and then the other variables as shown. The patient in **d**, on the other hand, had a predicted probability of length of stay of 0.06 or roughly one-fourth of the baseline, despite being admitted to the ICU within 24 h of admission. The major contributor to this low probability was the diagnosis of antidepressant poisoning, followed by a private insurance provider, and finally by a lack of BMI recorded in the chart for this encounter. The reasoning behind the importance of a missing value for BMI is unclear but is repeatedly apparent in several analyses and may have to do with systematic recording practices within the hospital system (see Agniel et al.^19^ for an exploration of this phenomenon).

LOS was predicted in terms of the number of days and was binarized at various thresholds. LOS in days was predicted poorly, within 3.97 days measured by root mean square error (RMSE; average LOS 2.94–3.71 days). LOS over 5 days was predicted with an AUC of 0.84 (Fig. 2a) and a BSL of 0.15 (calibration curve shown in Supplementary Fig. 1d). Average precision was 0.70 (see Supplementary Fig. 2d). When trained and evaluated on a cohort of 300,000 patients, performance was similar: AUC 0.81 and BSL 0.17. Other ML models, including a deep neural network, were trained on the same task, with performance generally inferior to the GBM (see Supplementary Fig. 2 and Supplementary Table 1). The most impactful features included the type of admission, primary diagnosis code, patient age, admission source, LOS of the most recent prior admission, medications administered in the hospital in the first 24 h, insurance, and early admission to the intensive care unit, among others shown in Fig. 2c, d. Impactful features for LOS at thresholds of 3 and 7 days are shown in Supplementary Fig. 5c, d, respectively. The AUC did not differ in these time points compared to 5 days (Table 2). Given that primary diagnosis is often assigned late in the hospital encounter or even after discharge, we trained the LOS models with and without this feature for comparison. Results are shown in Supplementary Table 1d. Overall, predictive performance was decreased, as expected. AUC for LOS > 5 days was 0.781, BSL was 0.173, and average precision was 0.640.

Prediction of death within 48–72 h of admission was predicted with an AUC of 0.91 and BSL of 0.001 (Table 2). However, owing to extreme class imbalance (e.g., in the testing set there were 260,518 non-deaths and 390 deaths), this was achieved by predicting non-death in every case. Strategies to produce a reliable model by addressing class imbalance, such as data oversampling, were unsuccessful. AUC and BSL do not reliably indicate model performance and applicability in this clinical setting.

### Variable interactions

SHAP analysis also allows examination of interactions between variables. Key variable interactions are shown in Supplementary Figs 6 and 7. For example, high and low values of heart rate were shown to affect probability of readmission differently for patients at different ages. With older patients, there is a clearer trend toward lower heart rates on discharge contributing to lower readmission risk and higher heart rates contributing to higher readmission risk, though modestly (SHAP values from −0.1 to +0.1–0.2). With younger patients, higher discharge heart rates overall are observed, and the positive trend is more modest. This may highlight the importance of considering a variable such as heart rate in a more complete clinical setting, such as one that includes patient age and clinical reasoning (e.g., an adult is unlikely to be discharged with marked tachycardia) (Supplementary Fig. 6c). A similar finding is observed in Supplementary Fig. 7c for LOS prediction, though clinical reasoning is less likely to play a role compared with more purely physiologic phenomena: higher heart rates overall are observed for pediatric patients, and the relationship between heart rate and LOS is not observed to be as linear for pediatric patients (high and low SHAP values are observed more uniformly for given levels of tachycardia in pediatric patients).

## Discussion

Our investigation of ML methods for predicting and explaining inpatient outcomes was initiated as a result of increased focus on the costs and risks of inpatient stays in the United States and other countries, availability of complex data in the EHR, and the development of explainable predictive models. In addition, recent concerns over the impact of metrics such as readmission rates^4^ yield an opportunity to develop models that may be used to not only predict but also understand the components of risk and their interactions. We therefore sought to predict and understand current and future readmissions and the LOS during hospitalization.

Our models achieved comparable performance to the existing state of the art in the prediction of readmission and LOS but with more explainable models^11,12^. By using a model that accounts for non-linear interactions, we can flexibly predict outcomes across a large number of patients with many diagnoses and comorbidities. In addition to reporting AUC, which assesses performance across classification cutoffs, we show that our models are well calibrated when using raw probabilities, which may be more useful than binary classifications in many settings^13^. The most important components of the probability prediction for each patient can be examined, which would ideally lead to items that can be further studied, perhaps leading to quality improvement efforts (e.g., patients with a high number of emergency department visits contributing significantly to their risk of readmission may be targeted for hotspotting efforts rather than the usual scheduled in-office follow-up)^14–17^ or at least to a deeper understanding of the current situation (e.g., a given diagnosis or necessary therapeutic agent may be associated with a higher risk of readmission or another adverse outcome, but these features are not likely modifiable)^18^. We also generate cohort-level diagrams that explain the contributions of each variable to the model output as well as key variable interactions.

Because of the focus on interpretability, the study was designed to cast a broad net with regards to inclusion criteria. Rather than including only CMS (Centers for Medicare and Medicaid Services)-defined readmissions, we chose to include all patients who survived the index hospitalization, including those in observation status. We also included all available diagnoses and ranges of demographic categories, including age. This allowed us to examine the impacts of these variables, as well as develop a broadly applicable model for the institution as a whole, which included many specialties, hospitals, and a range of socioeconomic environs. Using diverse data also allowed us to find interactions, such as the varying impacts of heart rate and number of administered medications on readmission risk across the range of ages. We also found, as have others^19^, that presence or missingness of data within the EHR can be informative on its own, as in the case of BMI measurement in Fig. 2c, d.

Our study is additionally unique for balancing a relatively simple model architecture and hand-selected variables with a robust and generalizable explanatory method. Rajkomar et al. achieved comparable results using a DL model trained on nearly 47 billion data points spread over ~215,000 patients, acquired with an automated data collection method^11^. Their explanatory method highlighted areas of the medical record that were most important for prediction but used restricted and less performant versions of their models, retrained on a single data type (text, laboratory results, etc.). Our approach is a direct interpretation of the full predictive algorithm and also explains the impact of variables across the range of possible values, rather than simply highlighting which variables were important. It may be the case that more highly tuned DL or other, less complex approaches would achieve similar or superior predictive power, but likely at the expense of either interpretability or richness^20–22^. It is also important to note that our approach and Rajkomar’s are not directly comparable, given the heavily specialized algorithms and explanatory methods used in their approach, with a different cohort, different data format, and breadth of variables considered. We used off-the-shelf algorithms that are free and open source, do not require advanced computational power, and may therefore be more accessible in less resource-rich settings. One of Rajkomar’s key contributions was the use of an interoperable, rich, dynamic data format, and hence their approach has an increased focus on the data pipeline proper, whereas ours is a more simple database query with a modest amount of feature engineering. However, we share the goal of predicting adverse outcomes with a high degree of explainability that targets decision support and hypothesis generation, rather than automated decision-making. Further, given the comparable performance metrics achieved by our approach and others in similar cohorts, it may be that the inherent complexity of readmissions and long LOS confer a natural upper limit on predictive power, encouraging a further focus on interpretability.

The study has several limitations. First, we selected only variables available at the beginning and end of the hospitalization. Second, because we only used data available in our EHR, we could only assess for readmissions with reference to our hospital system. We therefore did not capture the total readmission rate, nor could we account for admissions to our system that were readmissions from another system. Third, this was a retrospective study based on data from a single health system. It therefore requires external validation, though the most important variables that impacted each outcome were also described as important prognostic factors in prior reports, which suggests that our model could be applicable in other systems. Fourth, primary diagnosis code was used as a predictor. This is typically not available until some time after the encounter has completed and financial teams have processed the hospitalization and so would not be available for either LOS or readmission predictions in a live system. We are exploring ways to dynamically assign primary diagnosis within an encounter for our in-house implementations of the model, such as ranking the electronic medical record problem list according to surrogate markers of severity. Finally, and in summary, as with all ML seeking to explore causal relationships, this is a hypothesis-generating work, in need of rigorous validation, independent studies on promising components, and, ultimately, patient and clinician judgment as regards application. We hope that an emphasis on intelligence augmentation, decision support, and explainability will lead to a more nuanced and skilled adoption of ML as yet another tool in a holistic approach to patient care and research.

In conclusion, we generated prediction models that reliably predict the probability of readmission and LOS, which are explainable on the patient level and cohort level. We propose the use of this approach as an auditable decision aid that also contributes to hypothesis generation.

## Methods

### Data collection

Hospitalizations with a discharge date from January 2011 to May 2018 were extracted from the Cleveland Clinic (CC) EHR. Clinical, demographic, and institutional features were extracted using natural language processing and parsing of structured data available within the EHR (see Supplementary Table 2). Data available at the time of hospitalization (i.e., within roughly 24 h of encounter creation) and discharge were marked as such and used as appropriate to the predictive task. Publicly available American Community Survey census information was retrieved for each patient’s census block group (BlockGroup), which is based on home address and reports aggregate sociodemographic data for a small geographic region^23^. This study was approved by the CC Institutional Review Board with a waiver of individual informed consent due to the retrospective nature of the study and conducted in accordance with the Declaration of Helsinki.

The cohort of hospitalized patients was split into three groups for analysis: 80% for model development, 10% for testing, and 10% for validation. Selection of hospitalizations for inclusion in each group was random with the exception of ensuring that the rate of the positive class (30-day readmission, LOS over 5 days, etc.) was consistent between sets.

### Predictive modeling

GBM algorithms were used to produce predictive models. GBMs are nonparametric methods that train many decision trees in succession, using information from each set to optimize the performance of the next iteration^24^. GBMs achieve state-of-the-art performance in relation to other ML methods, especially in structured data^25^. They also allow for inclusion of many types of variables, and can explicitly account for missing data, and thus do not require imputation of missing values. More information regarding the GBM algorithm is available in Supplementary Materials. To reduce model overfitting, we employed a standard train/test/validation split and early stopping at 200 iterations^26,27^. For comparison, we also trained a deep neural network, logistic regression, and several other ML algorithms on the same data, applying standard imputation and scaling techniques. We performed ten-fold ten-repeat cross-validation to generate confidence intervals. Given that primary diagnosis is often not assigned until after the hospital encounter, we trained the LOS models with and without this feature for comparison. Finally, we trained our final model on a smaller subset of 300,000 hospitalizations to examine the effect of training data size on model performance.

### Model interpretation

To extract important variables that impacted the algorithm and ensure the appropriateness of the final models, cohort and personalized model predictions were interpreted using SHAP values^28^. SHAP values, based on the Shapley value from coalitional game theory, are consistent and accurate calculations of the contributions of each feature to any ML model’s prediction. They are additionally able to account for feature interactions, including situations where a given value may either increase or decrease risk (for example, a child with a heart rate of 130 vs. a geriatric patient with the same heart rate). SHAP values also overcome limitations inherent to standard variable importance information available in tree-based models, which yields an ordering of all variables used in the model by how much each impacts the predictions overall, by showing the impact of variables across the range of their values, the interactions of variables with each other, and allowing for case-specific (here, patient-specific) explanations as well as cohort-level exploration. More details regarding the SHAP package are summarized in Supplementary Materials.

### Statistical analysis

Descriptive statistics were used to summarize the patient cohort in general and in each subgroup. Model performance was assessed with metrics appropriate to the prediction endpoint. For binary outcomes, the BSL, AUC, and area under the precision-recall curve (average precision) were calculated. We also produced appropriate figures for these metrics, including calibration curves, which show the quality of a model’s proposed probability by comparing it with the percentage of patients at that probability with the outcome of interest (i.e., proposed probability vs. actual probability). Numeric outcomes including LOS in days and days until readmission were evaluated with RMSE. All analyses were performed with ScikitLearn v0.20.31^29^ and Python v3.6.6. More details regarding the statistical methods are summarized in Supplementary Materials.

### Reporting summary

Further information on research design is available in the Nature Research Reporting Summary linked to this article.

## Supplementary information

Supplementary Information Material

Reporting Summary Checklist

## Data Availability

The data that support the findings of this study are available in a deidentified form from Cleveland Clinic, but restrictions apply to the availability of these data, which were used under Cleveland Clinic data policies for the current study, and so are not publicly available.

## Code availability

We used only free and open-source software. The software packages used are described in the “Methods” section.

## References

[1] Auerbach AD, Neinstein A, Khanna R (2018). Balancing innovation and safety when integrating digital tools into health care. Ann. Intern. Med..

[2] Cabitza F, Rasoini R, Gensini GF (2017). Unintended consequences of machine learning in medicine. JAMA.

[3] Sniderman AD, D’Agostino RB, Pencina MJ (2015). The role of physicians in the era of predictive analytics. JAMA.

[4] Wadhera RK (2018). Association of the Hospital Readmissions Reduction Program with mortality among Medicare beneficiaries hospitalized for heart failure, acute myocardial infarction, and pneumonia. JAMA.

[5] Bojarski, M. et al. End to end learning for self-driving cars. Preprint at https://arxiv.org/abs/1604.07316 (2016).

[6] Bobadilla J, Ortega F, Hernando A, Gutiérrez A (2013). Recommender systems survey. Knowledge-Based Syst..

[7] Silver D (2018). A general reinforcement learning algorithm that masters chess, shogi, and Go through self-play. Science.

[8] Gulshan V (2016). Development and validation of a deep learning algorithm for detection of diabetic retinopathy in retinal fundus photographs. JAMA.

[9] Coudray N (2018). Classification and mutation prediction from non–small cell lung cancer histopathology images using deep learning. Nat. Med..

[10] Esteva A (2017). Dermatologist-level classification of skin cancer with deep neural networks. Nature.

[11] Rajkomar A (2018). Scalable and accurate deep learning with electronic health records. NPJ Digital Med..

[12] Artetxe A, Beristain A, Grana M (2018). Predictive models for hospital readmission risk: a systematic review of methods. Comput. Methods Prog. Biomed..

[13] Steyerberg EW (2010). Assessing the performance of prediction models: a framework for some traditional and novel measures. Epidemiology.

[14] Donzé J, Aujesky D, Williams D, Schnipper JL (2013). Potentially avoidable 30-day hospital readmissions in medical patients: derivation and validation of a prediction model. JAMA Intern. Med..

[15] Leppin AL (2014). Preventing 30-day hospital readmissions: a systematic review and meta-analysis of randomized trials. JAMA Intern. Med..

[16] Burke RE (2017). The HOSPITAL score predicts potentially preventable 30-day readmissions in conditions targeted by the hospital readmissions reduction program. Med. Care.

[17] Auerbach AD (2016). Preventability and causes of readmissions in a national cohort of general medicine patients. JAMA Intern. Med..

[18] Saunders ND (2015). Examination of unplanned 30-day readmissions to a comprehensive cancer hospital. J. Oncol. Pract..

[19] Agniel D, Kohane IS, Weber GM (2018). Biases in electronic health record data due to processes within the healthcare system: retrospective observational study. BMJ.

[20] Aubert CE (2017). Simplification of the HOSPITAL score for predicting 30-day readmissions. BMJ Qual. Saf..

[21] Garrison GM, Robelia PM, Pecina JL, Dawson NL (2017). Comparing performance of 30-day readmission risk classifiers among hospitalized primary care patients. J. Eval. Clin. Pract..

[22] Sun, C., Shrivastava, A., Singh, S. & Gupta, A. Revisiting unreasonable effectiveness of data in deep learning era. In *Proceedings of the IEEE International Conference on Computer Vision* 843–852 (IEEE, 2017).

[23] US Census Bureau. American community survey 5-year estimates, https://data.census.gov/cedsci/table?q=United%20States&tid=ACSDP5Y2015.DP05 (2015).

[24] Natekin A, Knoll A (2013). Gradient boosting machines, a tutorial. Front. Neurorobotics.

[25] Ke, G. et al. Lightgbm: a highly efficient gradient boosting decision tree. in *Advances in Neural Information Processing Systems* 3146–3154 (Neural Information Processing Systems Foundation, Inc., 2017).

[26] Hastie, T., Tibshirani, R. & Friedman, J. *The Elements of Statistical Learning: Data Mining, Inference, and Prediction* (Springer Science & Business Media, 2009).

[27] Zhang T, Yu B, others. (2005). Boosting with early stopping: convergence and consistency. Ann. Stat..

[28] Lundberg, S. M. & Lee, S.-I. A unified approach to interpreting model predictions. in *Advances in Neural Information Processing Systems 30* (eds Guyon, I. et al.) 4765–4774 (Curran Associates, Inc., 2017).

[29] Pedregosa F (2011). Scikit-learn: machine learning in python. J. Mach. Learn. Res..

