# Supplementary material for: Personalized predictions of patient outcomes during and after hospitalization using artificial intelligence
Source: NPJ Digit Med. 2020 Apr 3;3:51. doi: 10.1038/s41746-020-0249-z (PMC7125114; doi:10.1038/s41746-020-0249-z)
Supplement: Supplementary file 1 — Supplementary Information Material [file 41746_2020_249_MOESM1_ESM.pdf]

Supplementary Materials

Contents

Supplementary Methods 36
Structuring electronic health record data 2
Inclusion Criteria and Outcome Definitions 3
Readmission 3
Length of Stay 3
Death 3
Machine Learning Models 4
Interpretation of the final model 5
Statistical analysis 6
Supplementary Figures 8
Supplementary Figure 1. ROC-AUC and calibration curves for readmission and extended length of stay 8
Supplementary Figure 2. Comparisons of model performances 9
Supplementary Figure 3. GBM Feature Importances 20
Supplementary Figure 4. Examples of Personalized Predictions, extended 22
Supplementary Figure 5. Top SHAP Features. 31
Supplementary Figure 6. SHAP Variable Interactions for 30 day readmission 33
Tables 36
Supplementary Table 1. Model Performance Comparisons 36
Supplementary Table 2. Categorized variables 39
References 44

Supplementary Methods

Structuring electronic health record data

Cleveland Clinic uses the Epic electronic health record (EHR) system. To generate our databases, statistical techniques including similarity calculations and fuzzy matching, are used to clean, parse, map and validate the raw EHR data. The raw data are extracted from both the EHR and other disparate data sources, mapped to discrete ontologies, cleaned and standardized, and finally deposited into a clinical research data repository. Approximately 185 tables from different data sources are condensed into 18 research-ready tables in the data repository. We utilize identifiers from the freely available Unified Medical Language System (UMLS) to map 6.8 million patient-related terms, as well as approximately half a million custom UMLS identifiers that include providers, locations, and their relationships. The Metathesaurus from the UMLS combines synonymous terms and codes from disparate medical vocabularies into concise terms and identifiers. Only 9% of columns in the data repository (approximately 1,000 data points per patient) do not utilize UMLS identifiers. These non-UMLS columns include patient identifiers, dates, and visit identifiers, which we manually queried for this project. Ultimately, there are approximately 32,000 discrete data elements per patient comprised of both UMLS and non-UMLS data. From this large collection, we selected variables to predict outcomes during hospitalization based on expert opinion and published literature, then extracted these into tables suitable for machine learning algorithms.[1] The total number of variables for each prediction task varied based on which features would be appropriate, ranging from 285 for prediction of 30-day readmission (including variables that would be available at the

beginning, during the duration, and at the end of the admission), to 171 for length of stay (including only variables that would be available during the first 24-hours of admission). See Supplementary Tables 1 and 2 for a list of the variables that were used overall (Supplementary Table 1) and for each predictive task (Supplementary Table 2).

Inclusion Criteria and Outcome Definitions

Readmission

Readmission was defined as any new Cleveland Clinic (CC) hospitalization starting 4 hours after any CCF discharge. For prediction of readmission, patients whose discharge disposition was “expired” were removed. Patients with an admission class of “observation” were retained, as it has been suggested that readmission reduction programs have resulted in an increased use of the observational setting.[2,3] The 4 hour cutoff removed patients who were simply transferring from one CCF department or hospital to another, and was selected based on histogram analysis of first-day readmissions.

Length of Stay

Length of stay was defined as the time between a given discharge date and admission date for each hospitalization. Only variables available within 24 hours of admission were considered with the exception of primary diagnosis code, and patients with an admission class of “observation” were removed.

Death

Death within 48–72 hours of admission was defined as a recorded EHR, Social Security, or Ohio Death Index death date, or a discharge disposition of “expired,” within the given time frame. Only variables available within 24 hours of admission were

considered with the exception of primary diagnosis code, and patients with an admission class of “observation” were removed.

Machine Learning Models

We used Gradient Boosting Machines (GBMs) to predict binary and numeric outcomes of interest. Gradient boosting machines function by consecutively training decision trees to predict the outcome of interest. Each consecutive tree learns from the ensemble of predictions that came before it, and attempts to minimize the error of the current prediction.[4] The GBM implemented by LightGBM contains several optimizations that allow it to obtain robust models quickly. The chief optimization is on-the-fly binning of continuous variables into discrete buckets, in order to allow for more straightforward splitting of the decision tree.[5]

As mentioned in the main text, Gradient Boosting Machines, and LightGBM in particular, allow for heterogeneous data input, including variables with a large number of categories, missing values, and zero values. They do therefore not require imputation, which is advantageous when the lack of a variable for a particular case is important (as in a patient who has never been admitted before and therefore has a “Length of stay of last admission” of “missing,” rather than “zero,” which may indicate something entirely different). Additionally, not every patient has the same set of labs drawn, and it would be inappropriate to impute values for these, especially if the imputation was based on the average or median value across the cohort, considering that many patients are likely to have abnormal values if the lab warranted being drawn. GBMs also do not require scaling, rendering the output of the explanations more human-readable. Lastly, because of the nonlinear combinations of variables probable

in a large healthcare dataset, a tree-based method such as GBM may be more interpretable than a linear model. This is primarily due to the likelihood of the latter to exhibit greater sensitivity to "model mismatch," wherein the high-bias nature of the linear model cannot adequately represent the underlying nature of the data, and so may be more likely to report spurious associations even at a comparable accuracy.[6]

We used Bayesian hyperparameter optimization, as available in the Python package hyperopt, to select hyperparameters for the main predictive targets.

We also used several comparator models, including a deep neural network within the Pytorch framework as implemented by fast.ai and several standard ML models from sklearn. Standard data imputation and scaling techniques were applied to the data to allow ingestion by the models.

Interpretation of the final model

The SHAP packages provides utilities for calculating Shapley values for a variety of machine learning algorithms, and is optimized for tree-based algorithms such as GBM. Shapley values come from classical game theory, and are the only additive feature attribution method that yield the combination of local accuracy, consistency, and allowance for missingness.[6,7]

SHAP values may be used to explain a model globally, by examining the average contribution of a given feature to the model output, or locally, by examining the most important variables for a given prediction.[7] They may also be used to examine interactions between variables. SHAP values were generated using the Python package shap v0.28.5. Visualizations were created using the Python package matplotlib v3.0.311, as well as the LaTeX typesetting language, as appropriate.

Statistical analysis

ROC curves, precision-recall curves, and calibration plots were generated for visual assessments of model performance. Additionally, summary values for these were calculated, including Brier scores for calibration plots, average precision for the precision-recall curves, and ROC AUC for ROC curves.

Calibration curves are used to assess the trustworthiness of a model's predicted probability. They provide a visual representation of the model's predicted probability vs. the fraction of samples at that probability with the actual outcome. The curve of perfectly calibrated model would exhibit a straight 45° line. Lower Brier scores are better, with a score <0.25 generally considered indicative of a useful model.[8] They are calculated as the mean squared difference between the probability assigned to each sample and the actual outcome (1 or 0). [8]

ROC curves, the corresponding ROC AUC, and precision-recall curves, with the average precision metric, show classification performance at all possible classification thresholds.[9] Higher numbers are better. ROC AUC and average precision of 0.5 indicate a model that performs no better than chance. An AUC of 1.0 indicates 100% true positive and 0% false positive rates, while an average precision of 1.0 indicates a positive predictive value of 100%. Confusion matrices show the number of samples correctly and incorrectly classified.

RMSE is calculated as the square root of the average of squared errors, or difference between observed and expected values, and yields a metric in the same units as the predictive target (here, days or years).[10] Median absolute error is the median absolute difference between the predicted target and the actual value, and

mean absolute error is the mean of the same. R2 scores are the percentage of the target variable variation captured by the model, where 100% indicates a model that explains all of the variability and 0% indicates a model that explains none.

Supplementary Figures

Supplementary Figure 1. ROC-AUC and calibration curves for readmission and extended length of stay

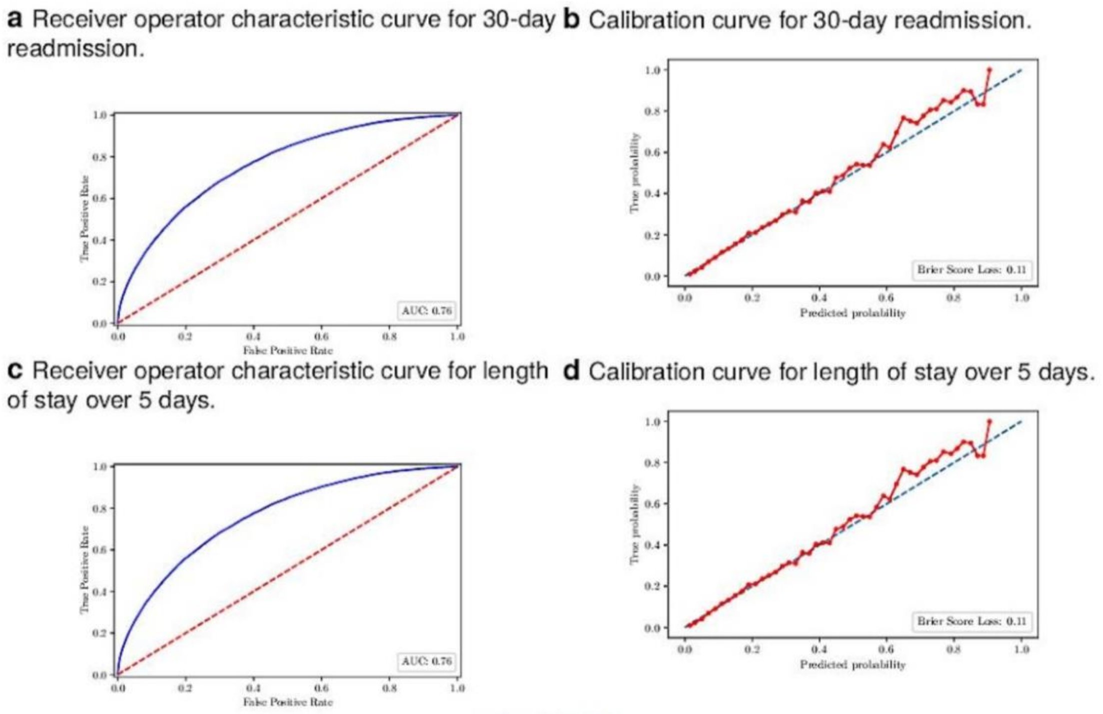

Supplementary Figure 2. Comparisons of model performances

a. Comparison of model calibration for 30 day readmission

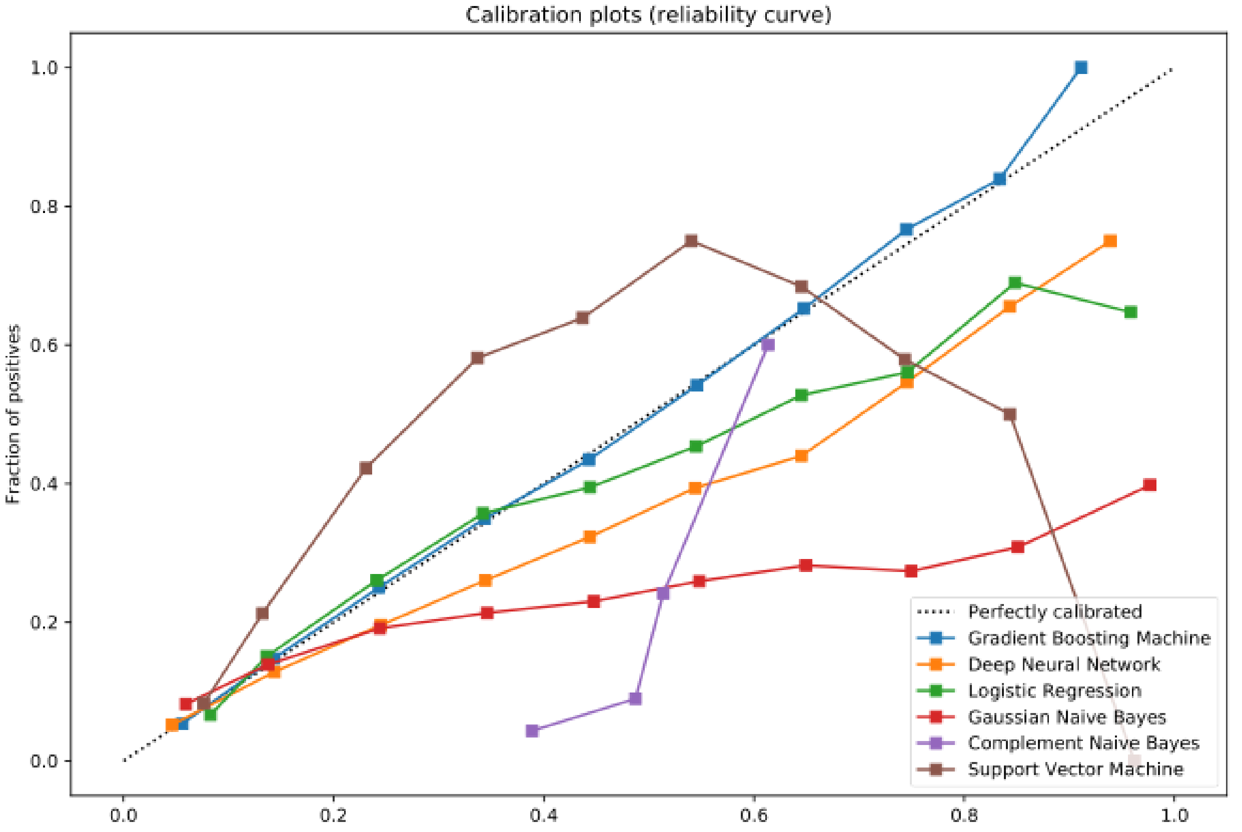

b. Comparison of model calibration for length of stay > 5 days

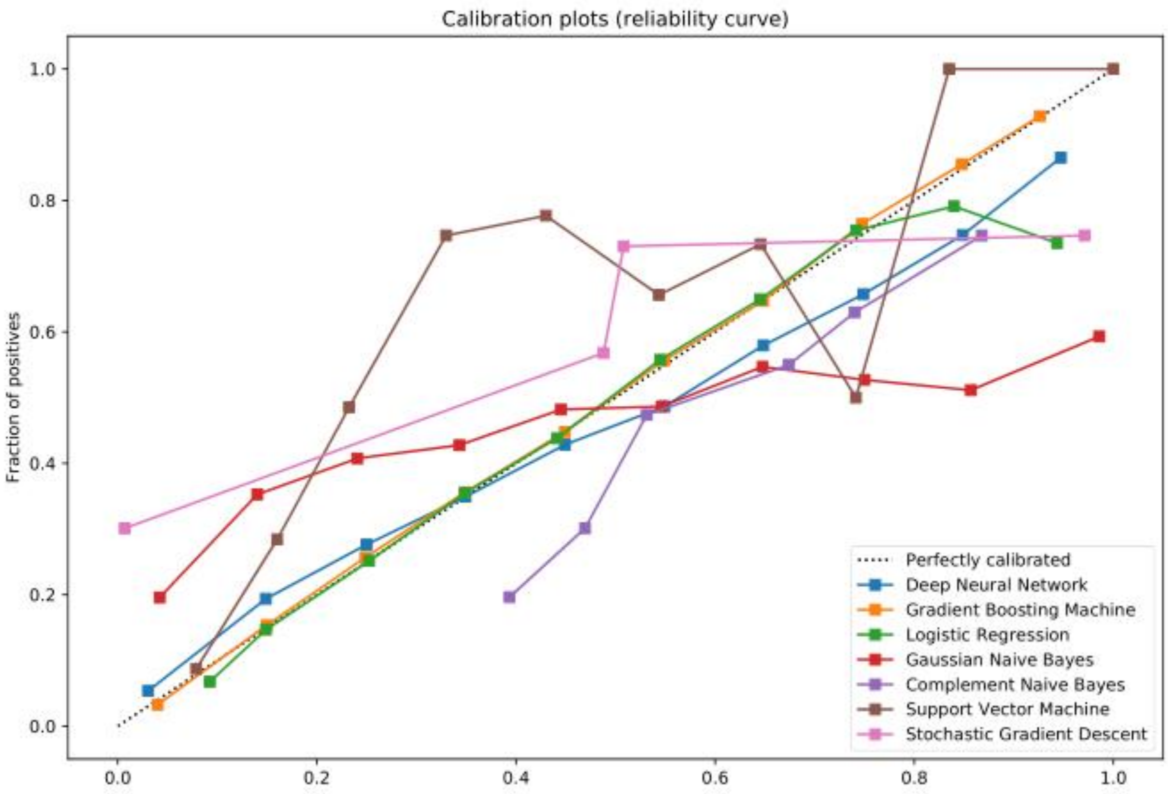

c. Precision-Recall Curve for 30 day readmission

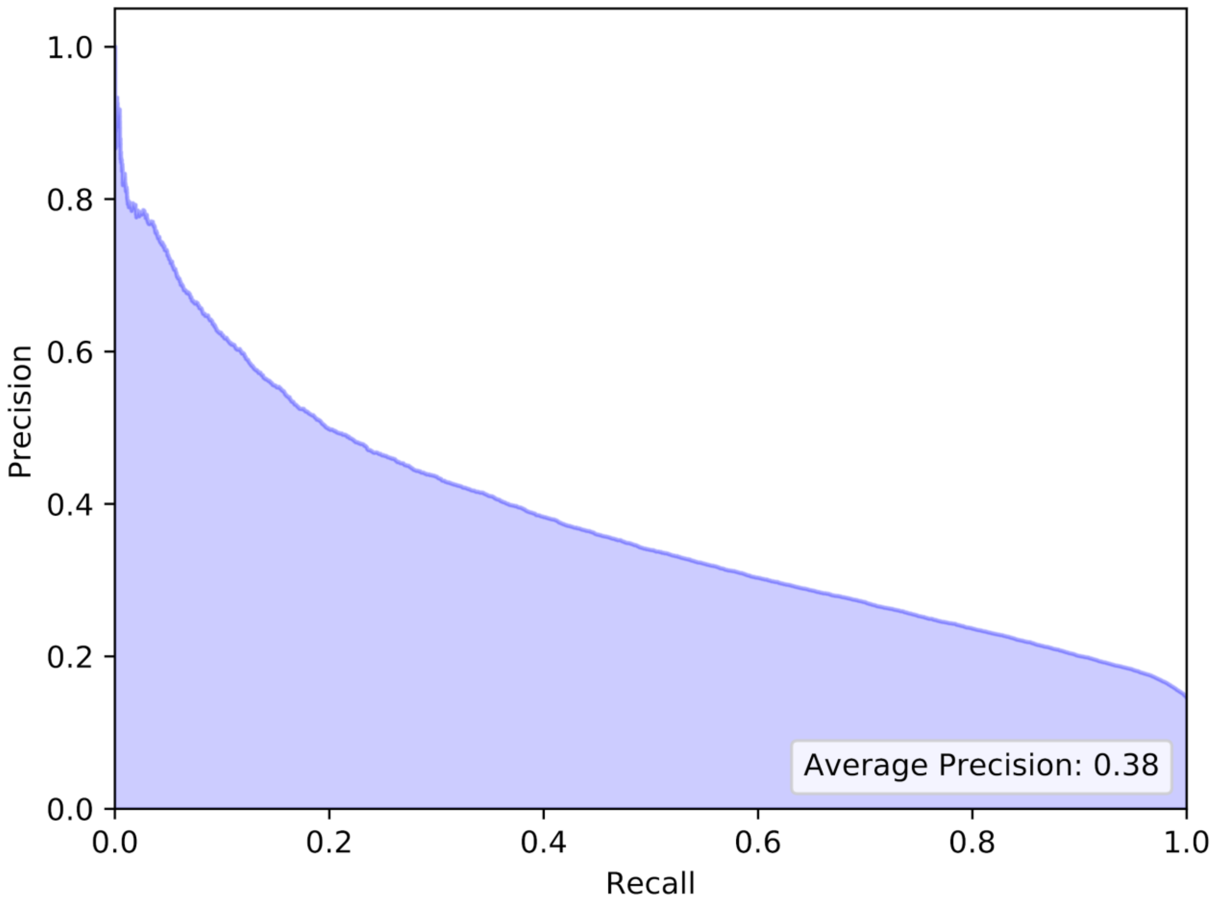

d. Precision-Recall Curve for length of stay > 5 days

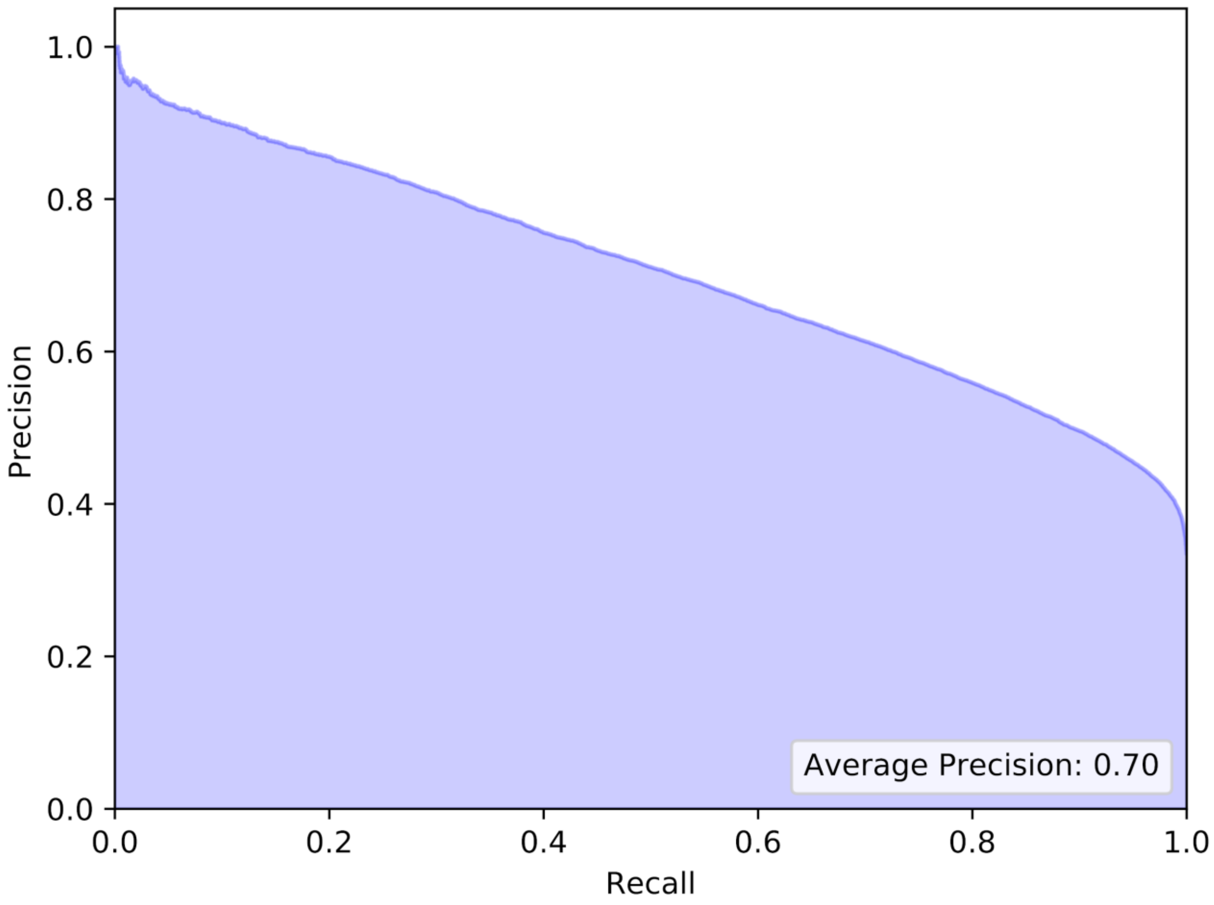

e. Extended model comparison figures for 30 day readmission (10x 10-fold cross validation)

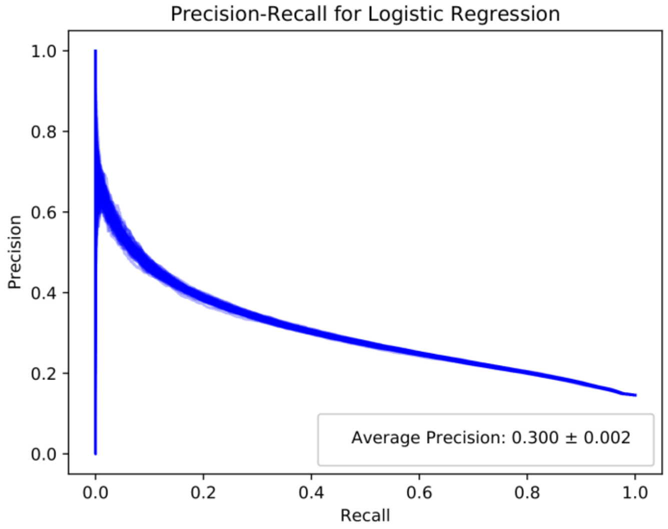

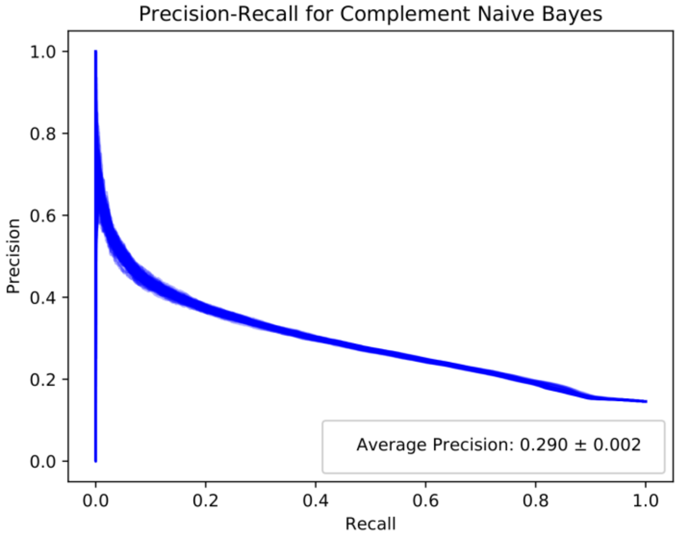

30d readmission

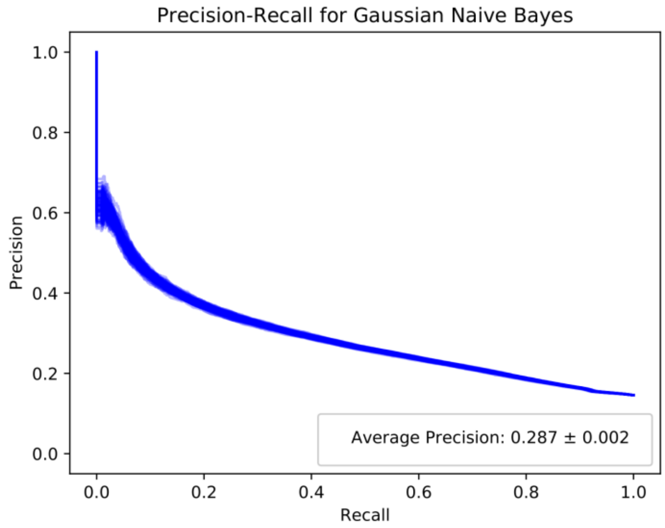

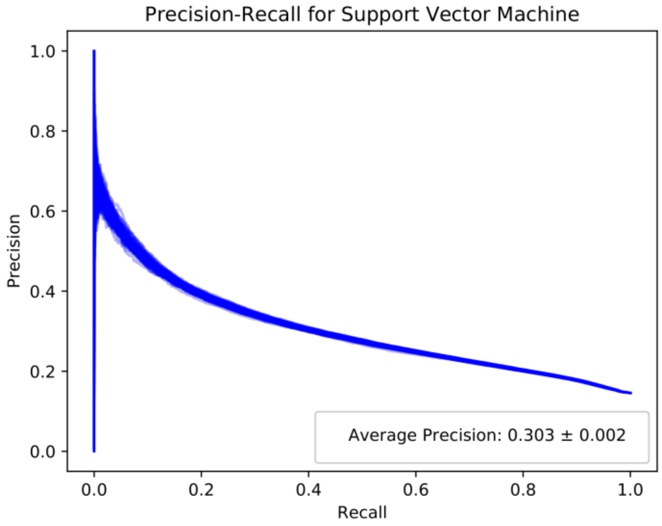

30d readmission

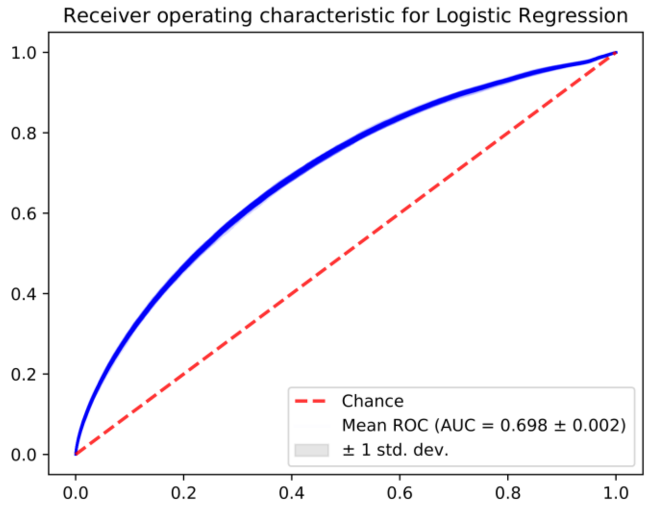

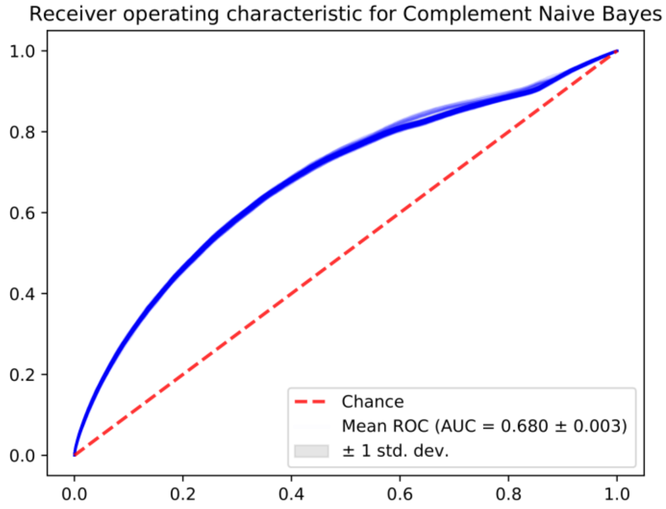

30d readmission

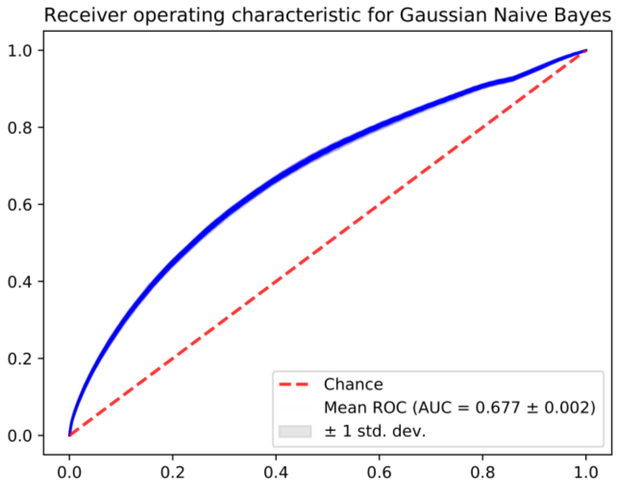

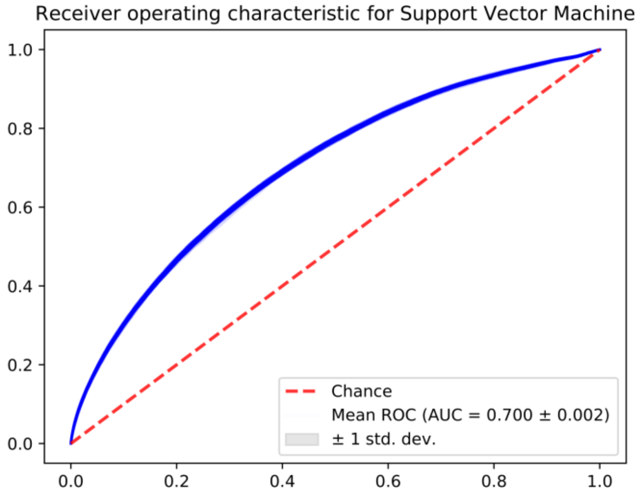

f. Extended model comparison for length of stay > 5 days (10x 10-fold cross validation)

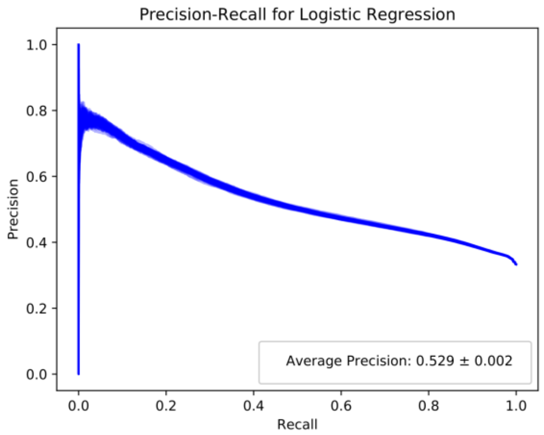

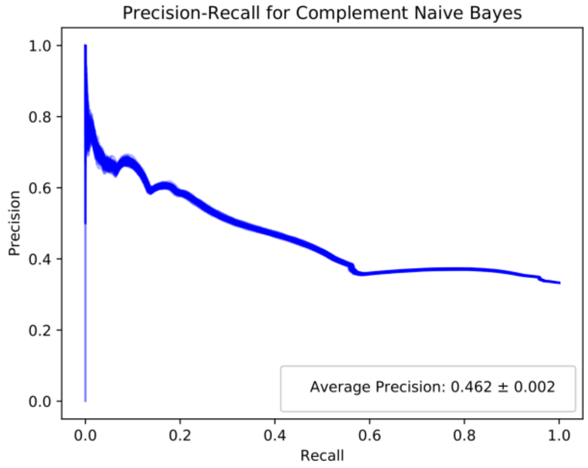

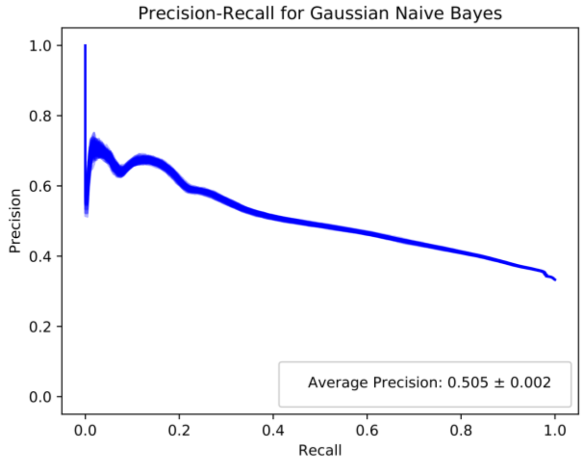

LOS > 5d

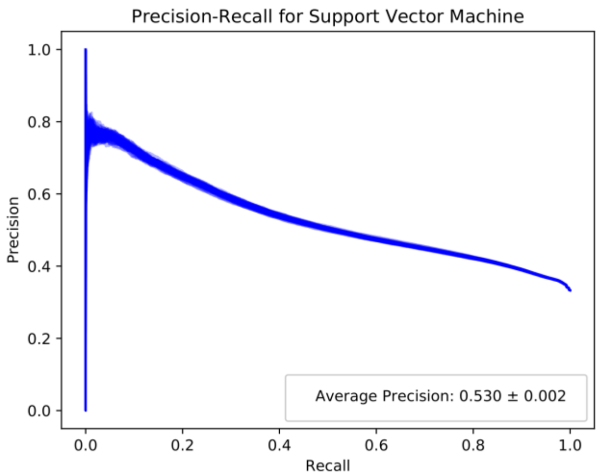

Precision-Recall for Support Vector Machine
Precision
Recall
Average Precision: 0.530 ± 0.002
0.0
0.2
0.4
0.6
0.8
1.0

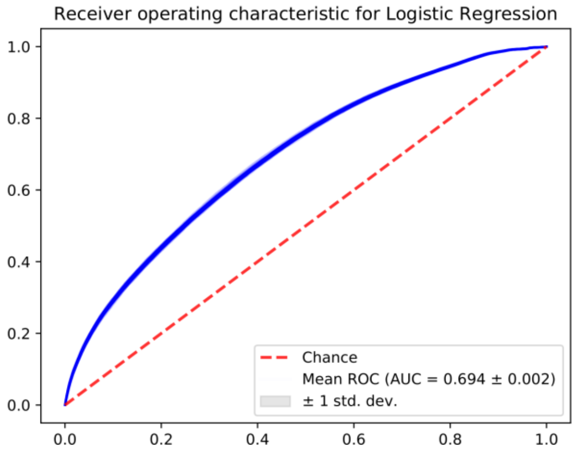

Receiver operating characteristic for Logistic Regression
Chance
Mean ROC (AUC = 0.694 ± 0.002)
± 1 std. dev.
0.0
0.2
0.4
0.6
0.8
1.0

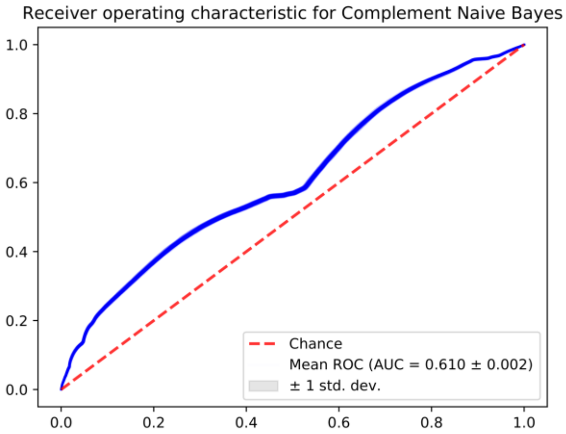

Receiver operating characteristic for Complement Naive Bayes
Chance
Mean ROC (AUC = 0.610 ± 0.002)
± 1 std. dev.
0.0
0.2
0.4
0.6
0.8
1.0

LOS >5d

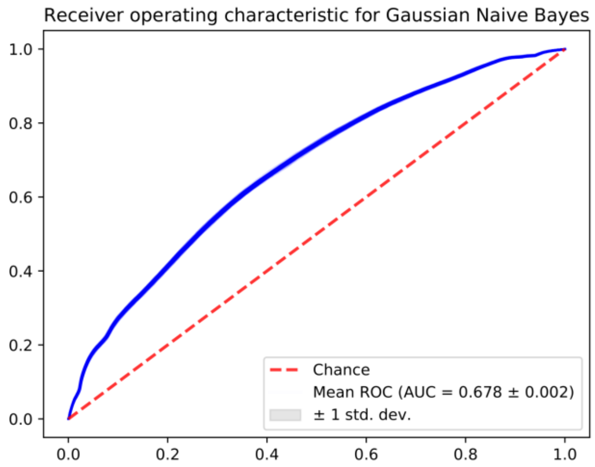

Receiver operating characteristic for Gaussian Naive Bayes
1.0
0.8
0.6
0.4
0.2
0.0
0.0
0.2
0.4
0.6
0.8
1.0
Chance
Mean ROC (AUC = 0.678 ± 0.002)
± 1 std. dev.

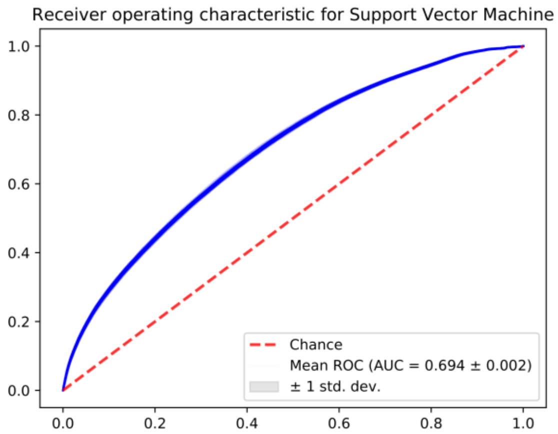

Receiver operating characteristic for Support Vector Machine
1.0
0.8
0.6
0.4
0.2
0.0
0.0
0.2
0.4
0.6
0.8
1.0
Chance
Mean ROC (AUC = 0.694 ± 0.002)
± 1 std. dev.

Supplementary Figure 3. GBM Feature Importances

a. GBM feature importance (not SHAP), 30 Day Readmission

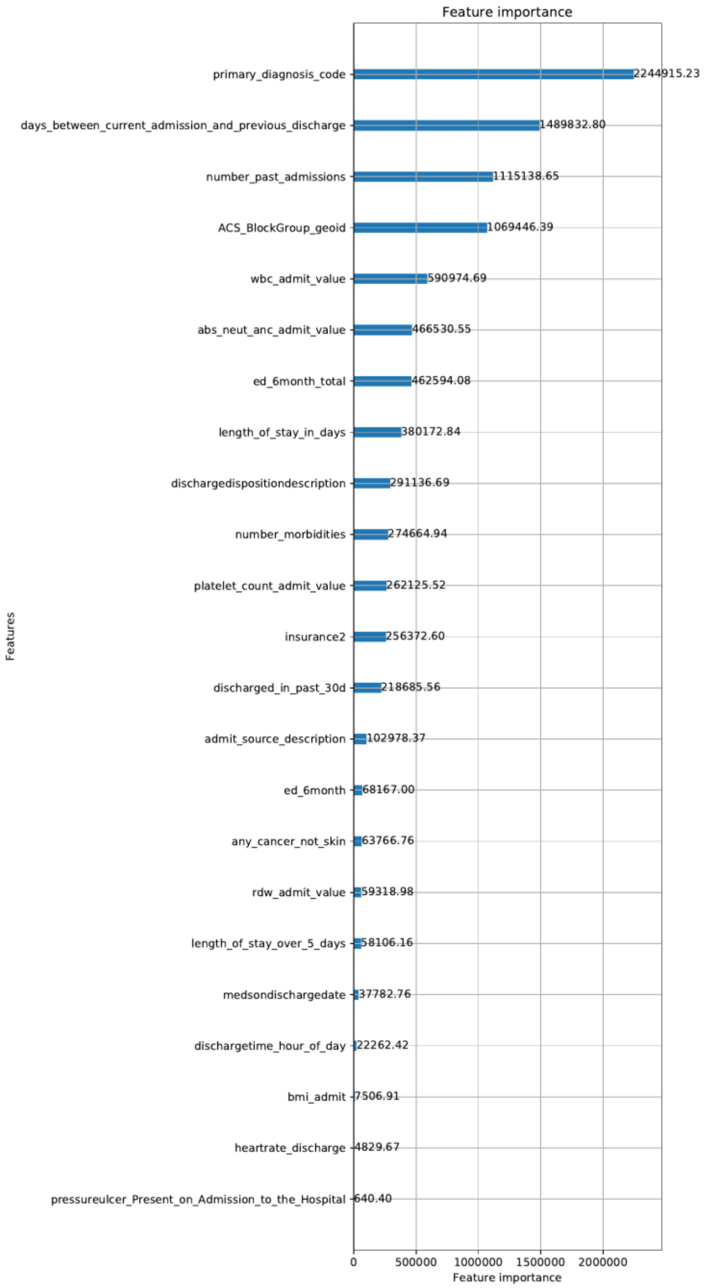

b. GBM feature importance (not SHAP), Length of Stay > 5d

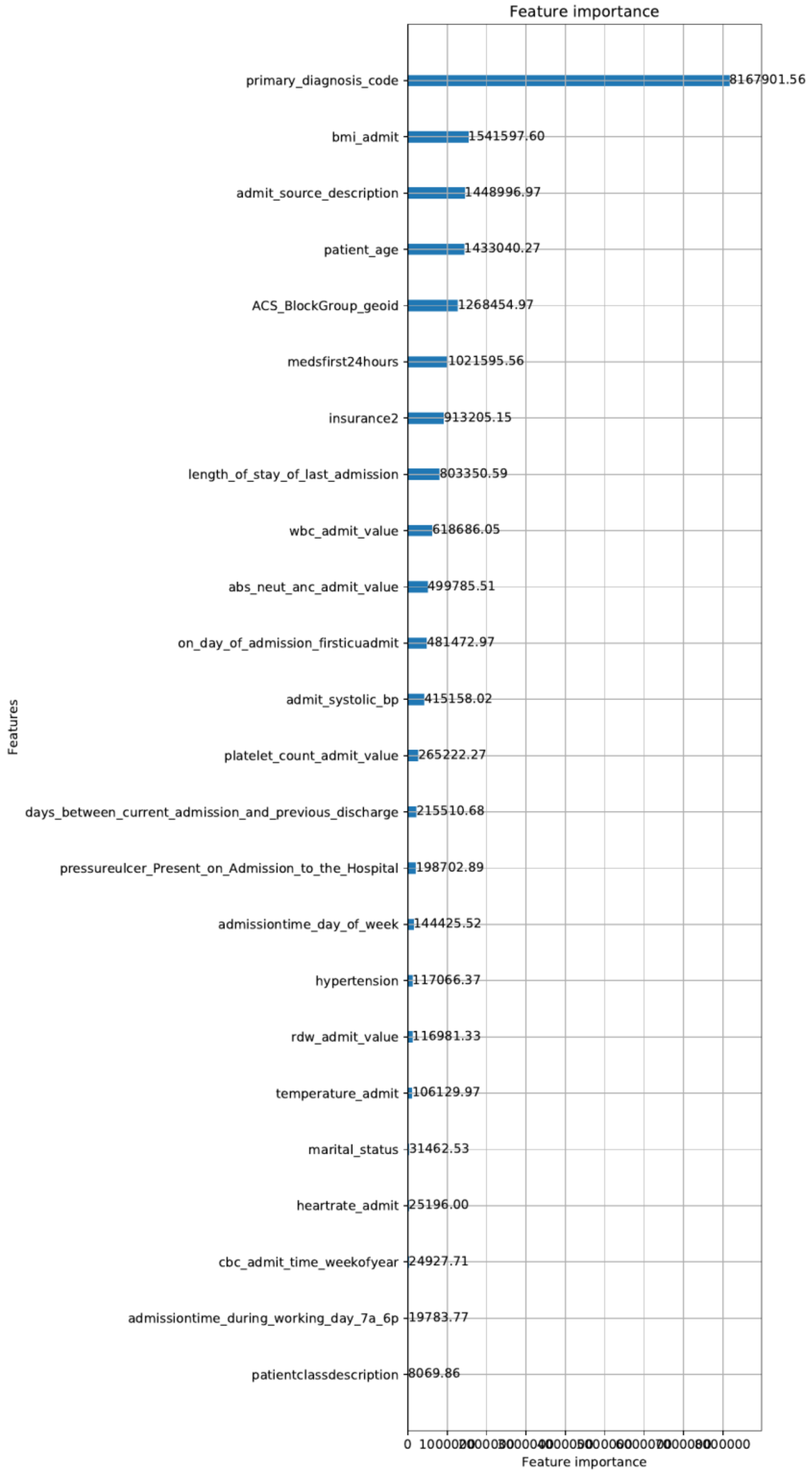

Supplementary Figure 4. Examples of Personalized Predictions, extended

a. Readmitted within 30 days

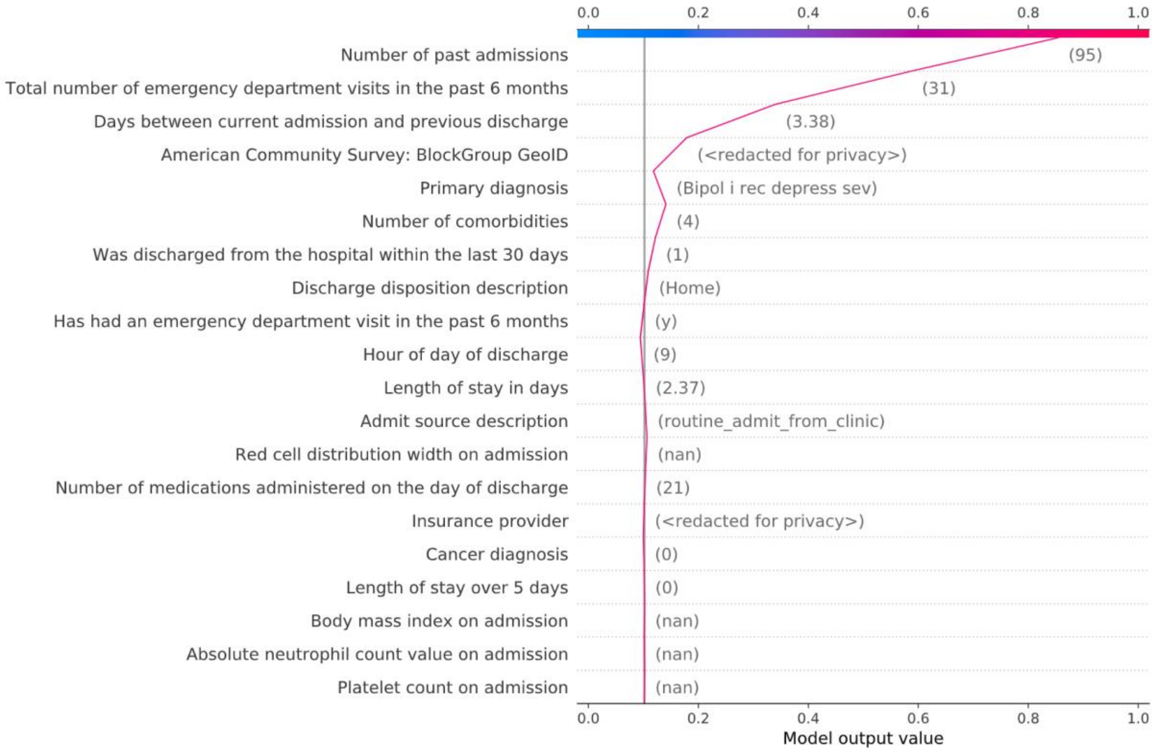

Pt with high probability of readmission within 30 days, largely due to significant history of admission and readmission, in addition to BlockGroup GeoID and other factors. Interestingly, the primary diagnosis (Bipolar disorder) decreased the likelihood of readmission.

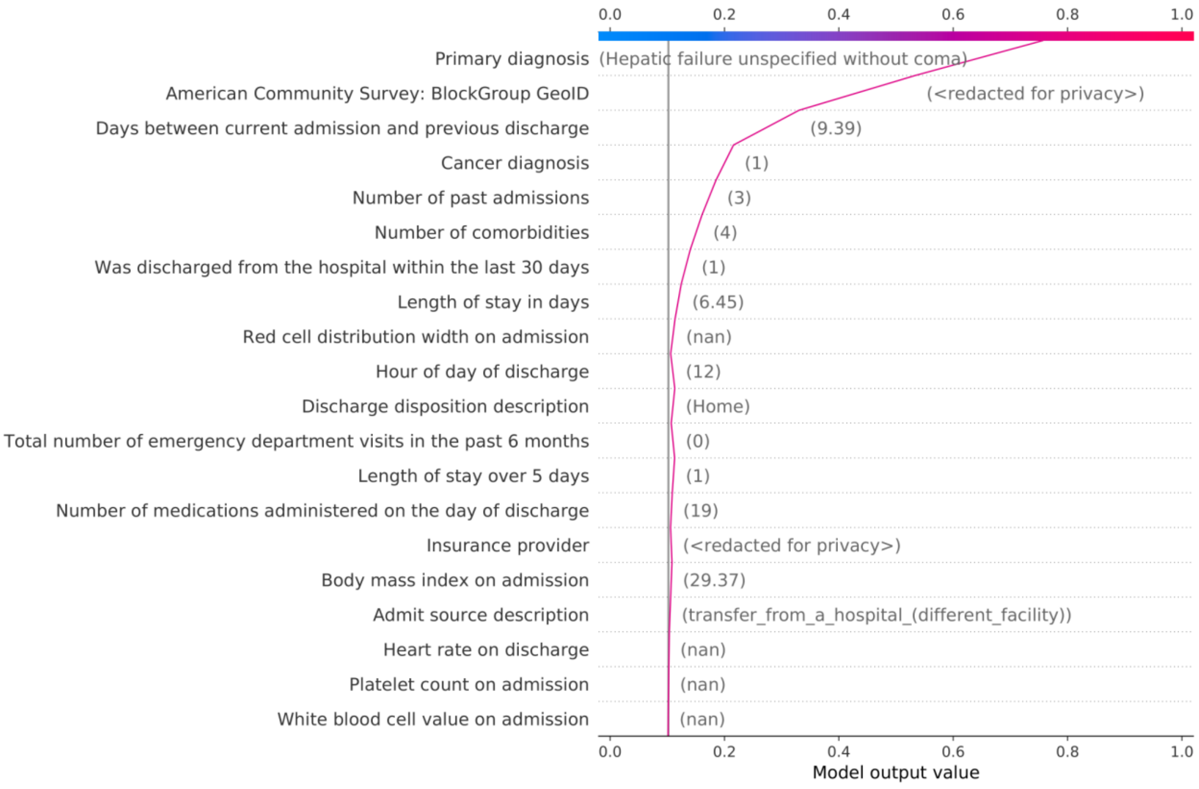

Another pt with high probability of readmission within 30 days, primarily due to a diagnosis of hepatic failure, in addition to their BlockGroup GeoID, recent admission, and cancer diagnosis. Number of past admissions played a role, but a less extreme one compared to the example above.

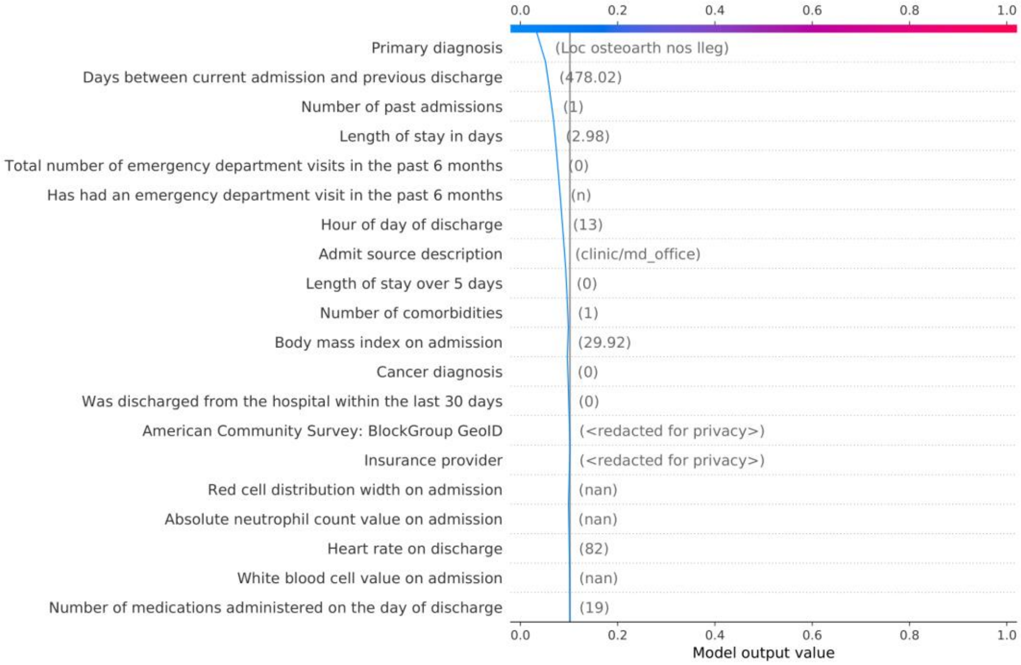

Pt with very low probability of readmission within 30 days, largely due to a diagnosis of osteoarthritis, a single prior admission that was over a year before the current admission, a short length of stay, and other variables as shown.

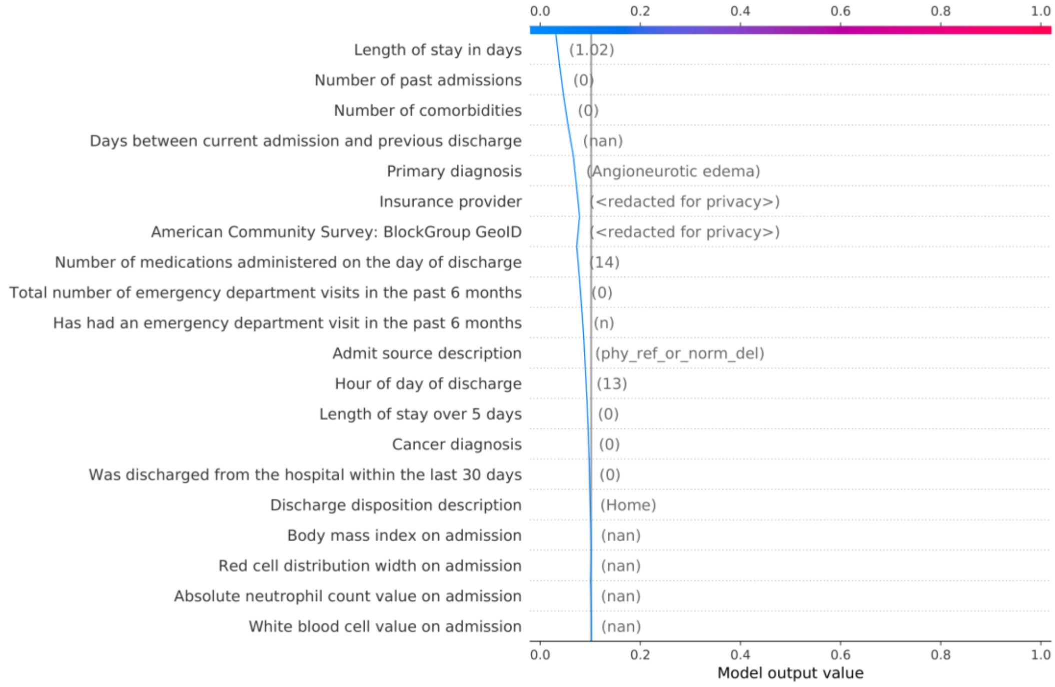

Another pt with very low probability of readmission within 30 days, largely due to a very short length of stay for angioneurotic edema, no prior admissions or listed comorbidities, and low number of listed medications on the day of discharge (all prescribed treatments are counted in this number).

b. Length of stay > 5 days

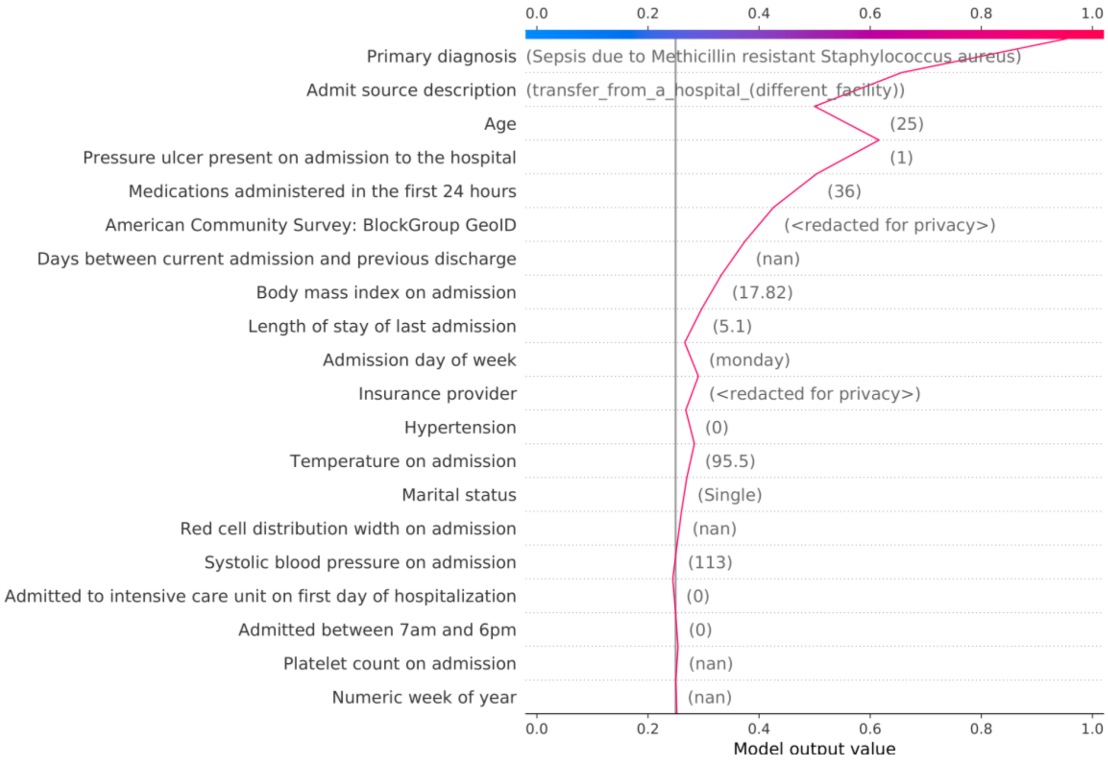

Young pt with MRSA-related sepsis transferred to our facility, with a nearly 100% probability of LOS >5d.

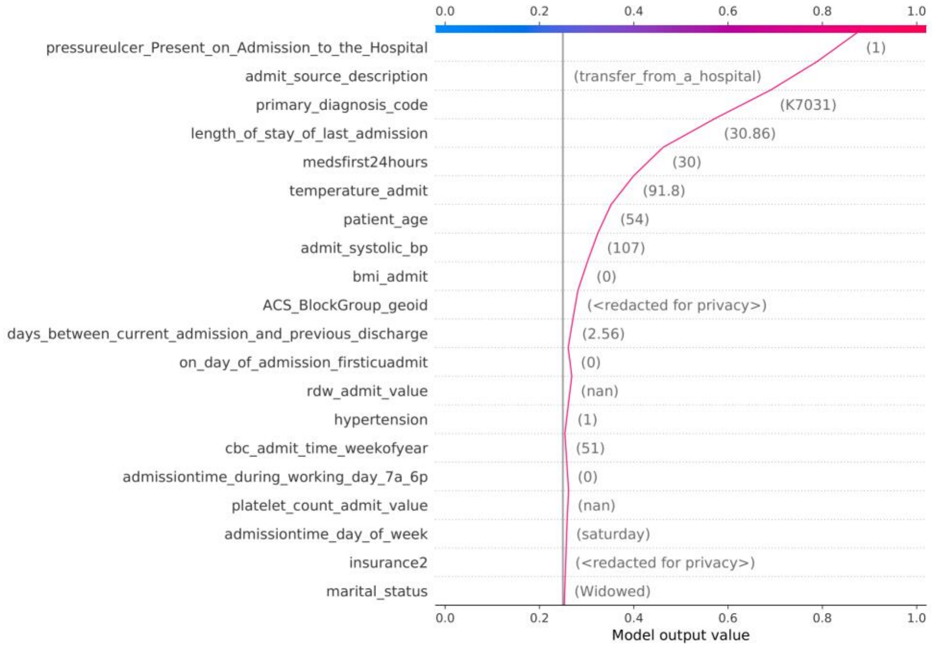

Pt with alcoholic liver cirrhosis (K7031) transferred to our hospital with a pressure ulcer and a lengthy prior admission, with a non-recorded BMI and low systolic blood pressure, but no ICU admission. Assigned ~90% probability of long LOS.

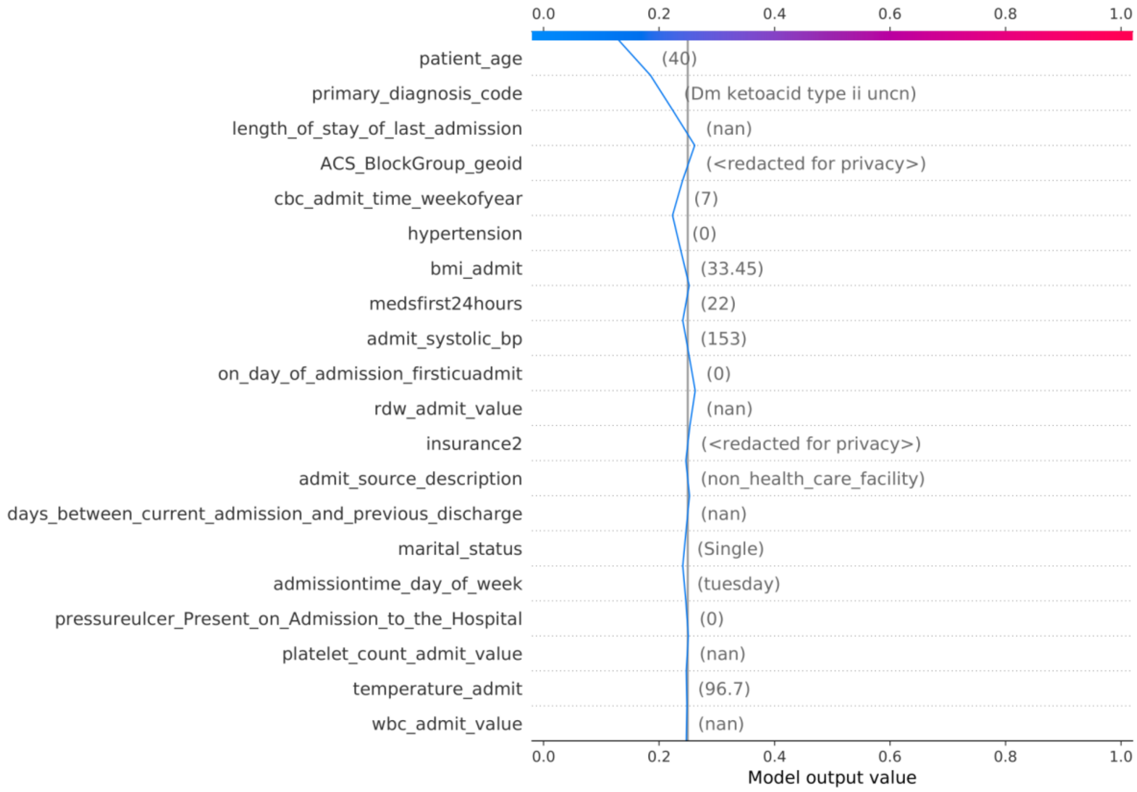

Relatively young pt with Type 2 Diabetes admitted for ketoacidosis, who had never been admitted before. Assigned a probability of LOS >5d ~15%.

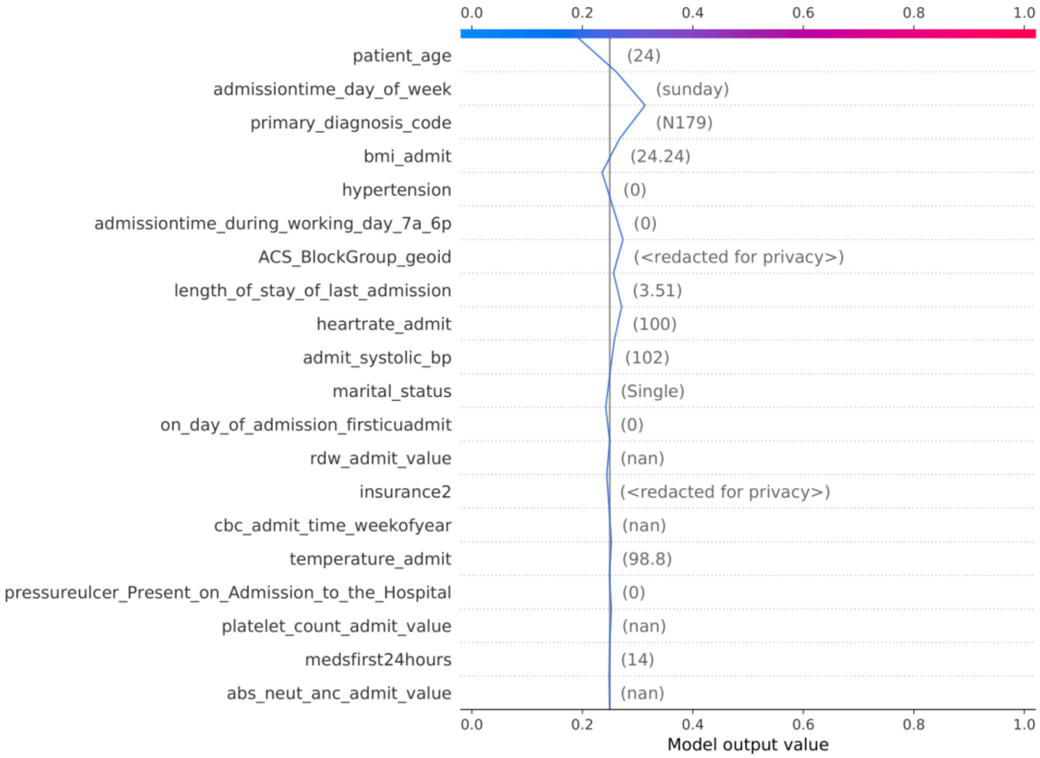

24 year old pt with primary diagnosis of acute kidney failure unspecified, admitted on a Sunday outside of normal working hours, with a probability of long LOS just under 20%.

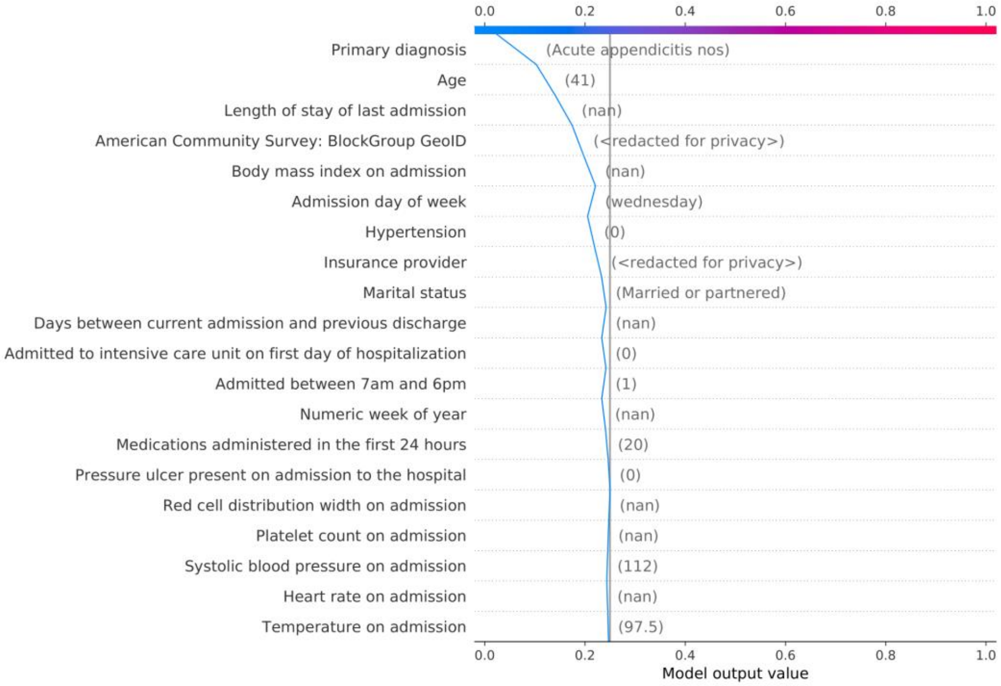

41 year old pt admitted on Wednesday during the working day for acute appendicitis, who had never had any recorded prior admissions and did not have BMI recorded at this admission.

Supplementary Figure 5. Top SHAP Features.

a. 3-day readmission

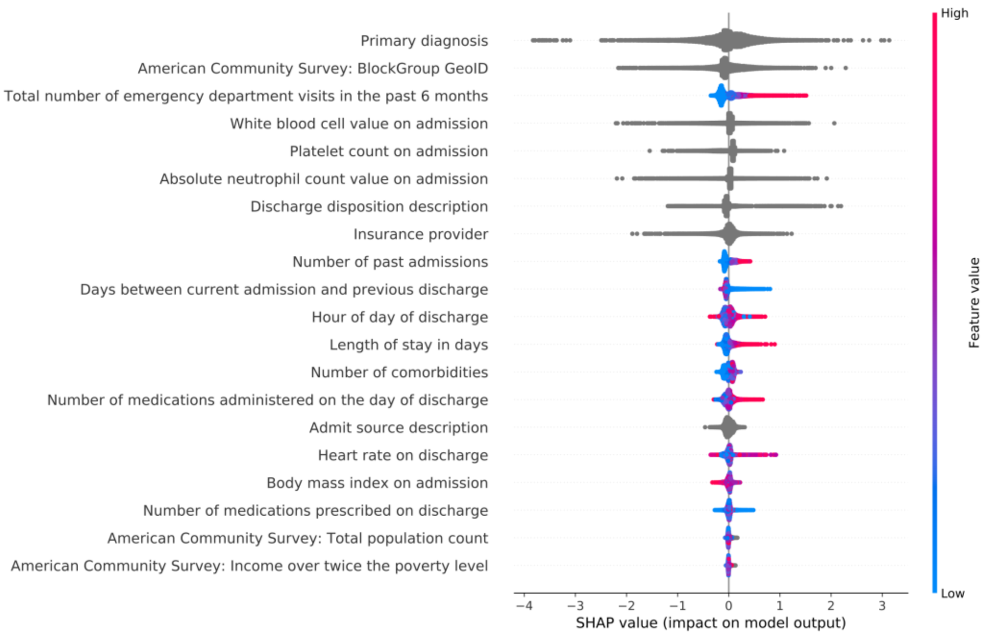

b. 7-day readmission

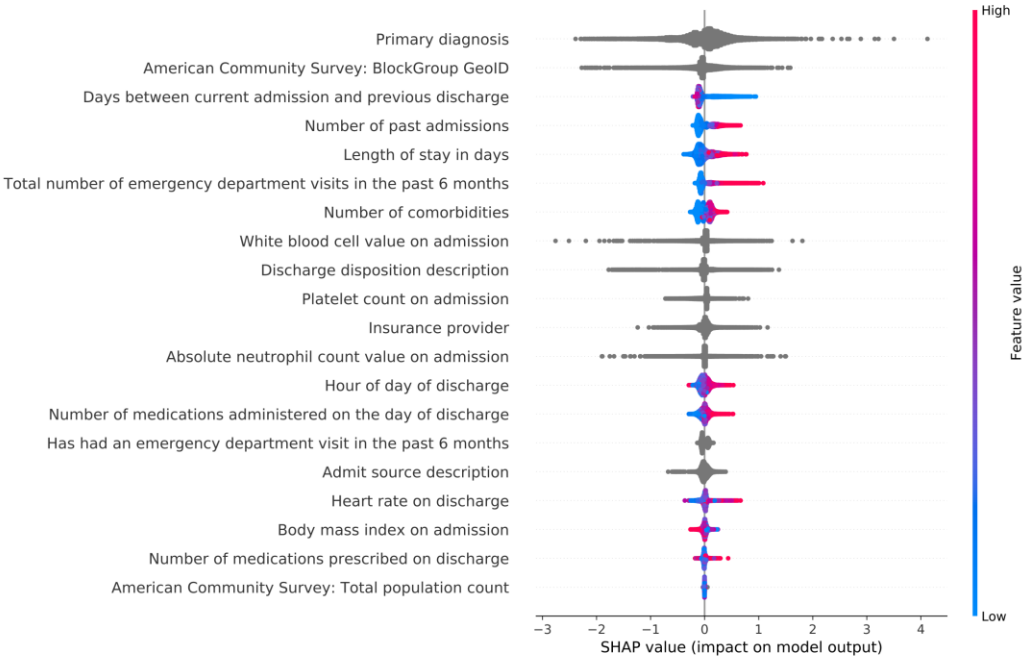

c. Length of stay over 3 days

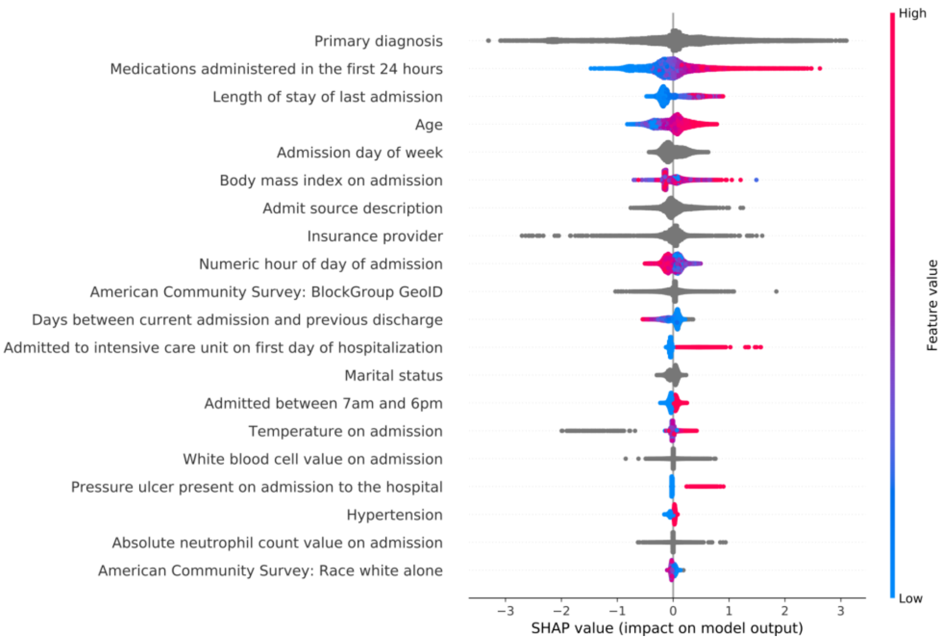

d. Length of stay over 7 days

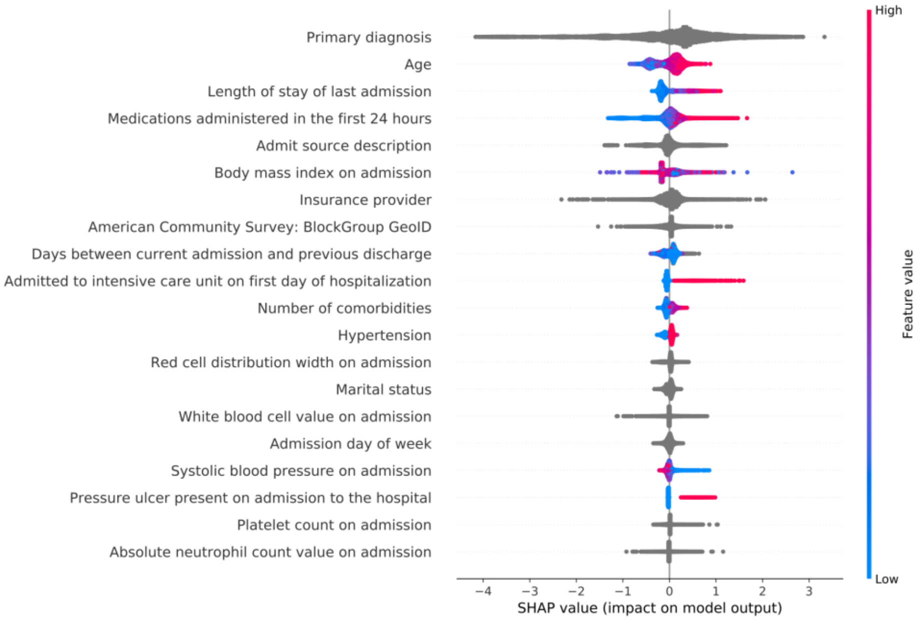

Supplementary Figure 6. SHAP Variable Interactions for 30 day readmission

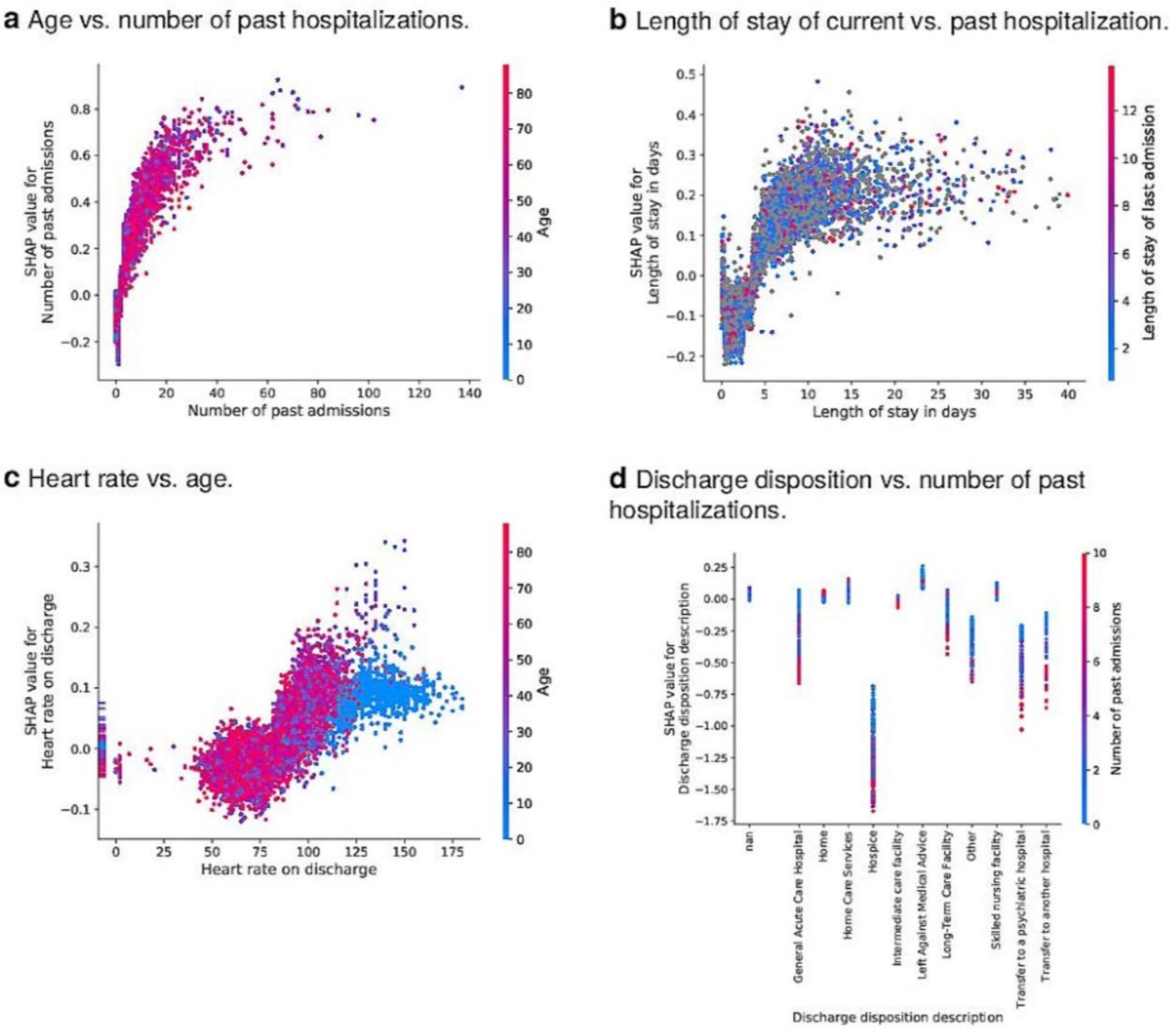

Supplementary Figure 7. SHAP Variable Interactions for length of stay > 5 days

a Number of medications administered in first 24 hours vs. age.

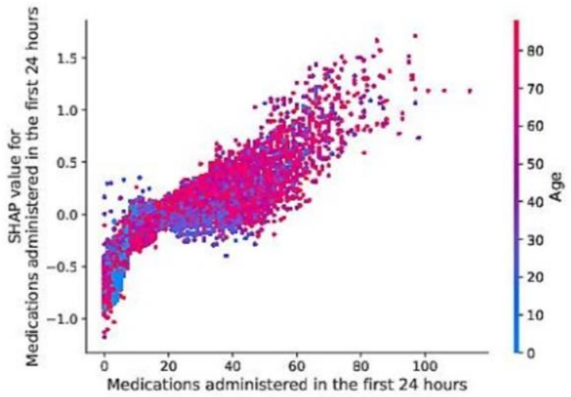

b Length of stay of last hospitalization vs. days since last hospitalization.

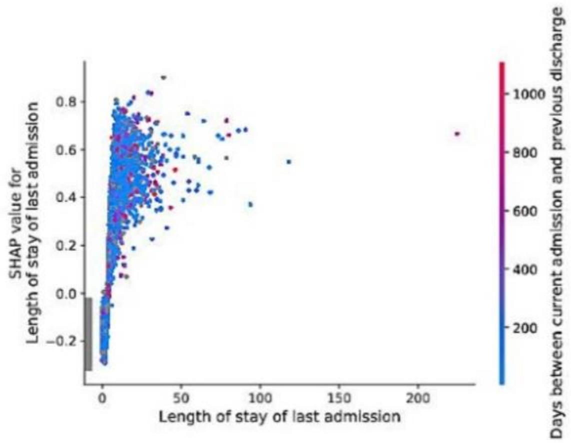

c Heart rate vs. age.

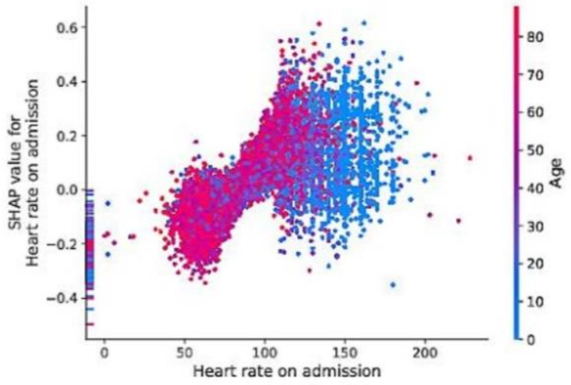

d ICU admission vs. number of medications administered in last 24 hours.

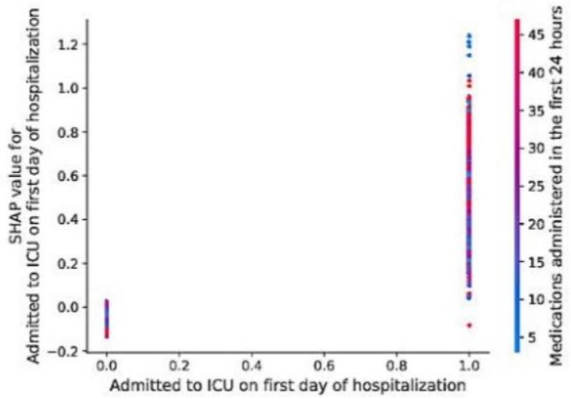

Tables

Supplementary Table 1. Model Performance Comparisons

a. 30 day readmission

Algorithm	Average Precision	ROC AUC	Precision	Recall	Accuracy	F1 Score	Matthews Correlation Coefficient	Brier Score Loss
Gradient Boosting Machine	0.383 [0.377-0.388]	0.758 [0.755 - 0.762]	0.632 [0.620-0.647]	0.102 [0.098-0.106]	0.861 [0.860-0.861]	0.176 [0.169-0.182]	0.214 [0.208-0.220]	0.108 [0.108-0.109]
Deep Neural Network	0.296 [0.292 - 0.300]	0.731 [0.728 - 0.733]	0.450 [0.445 - 0.454]	0.110 [0.105 - 0.113]	0.871 [0.869 - 0.873]	0.177 [0.173 - 0.182]	0.174 [0.171 - 0.178]	0.103 [0.100 - 0.108]
Logistic Regression	0.300 [0.296-0.305]	0.698 [0.694-0.702]	0.541 [0.521-0.565]	0.050 [0.047-0.053]	0.855 [0.855-0.856]	0.091 [0.087-0.097]	0.131 [0.124-0.140]	0.116 [0.115-0.116]
Complement Naive Bayes	0.290 [0.285-0.294]	0.680 [0.675-0.686]	0.242 [0.240-0.246]	0.615 [0.605-0.620]	0.664 [0.661-0.672]	0.348 [0.344-0.351]	0.210 [0.204-0.214]	0.245 [0.245-0.245]
Gaussian Naive Bayes	0.287 [0.282-0.291]	0.677 [0.672-0.680]	0.345 [0.333-0.373]	0.249 [0.202-0.269]	0.821 [0.816-0.834]	0.289 [0.262-0.302]	0.193 [0.185-0.201]	0.145 [0.140-0.147]
Support Vector Machine	0.303 [0.298-0.307]	0.700 [0.696-0.703]	0.591 [0.565-0.618]	0.032 [0.029-0.034]	0.856 [0.855-0.856]	0.060 [0.056-0.065]	0.112 [0.105-0.119]	0.123 [0.119-0.125]

b. Length of Stay > 5 days

Algorithm	Average Precision	ROC AUC	Precision	Recall	Accuracy	F1 Score	Matthews Correlation Coefficient	Brier Score Loss
Gradient Boosting Machine	0.704 [0.702 - 0.707]	0.828 [0.823 - 0.831]	0.692 [0.690 - 0.695]	0.542 [0.539 - 0.544]	0.767 [0.765 - 0.770]	0.608 [0.605 - 0.611]	0.452 [0.449 - 0.456]	0.156 [0.152 - 0.159]
Deep Neural Network	0.674 [0.669 - 0.677]	0.811 [0.807 - 0.814]	0.639 [0.634 - 0.644]	0.582 [0.577 - 0.585]	0.747 [0.742 - 0.752]	0.609 [0.604 - 0.612]	0.424 [0.421 - 0.428]	0.169 [0.166 - 0.173]
Support Vector Machine	0.530 [0.527-0.532]	0.694 [0.693-0.696]	0.636 [0.632-0.640]	0.221 [0.219-0.224]	0.698 [0.697-0.699]	0.328 [0.325-0.331]	0.233 [0.229-0.235]	0.270 [0.253-0.286]
Complement Naive Bayes	0.462 [0.460-0.465]	0.610 [0.608-0.612]	0.584 [0.581-0.588]	0.201 [0.198-0.202]	0.686 [0.686-0.687]	0.299 [0.296-0.300]	0.192 [0.190-0.194]	0.240 [0.240-0.240]
Logistic Regression	0.529 [0.527-0.531]	0.694 [0.693-0.695]	0.625 [0.624-0.629]	0.238 [0.236-0.239]	0.699 [0.698-0.699]	0.344 [0.343-0.346]	0.236 [0.235-0.238]	0.198 [0.198-0.199]
Gaussian Naive Bayes	0.505 [0.502-0.507]	0.678 [0.677-0.679]	0.581 [0.579-0.584]	0.260 [0.258-0.260]	0.691 [0.690-0.692]	0.359 [0.357-0.360]	0.220 [0.218-0.222]	0.250 [0.248-0.250]

c. Model comparisons, extended

Target	Algorithm	Average Precision	ROC AUC	Precision	Recall	Accuracy	F1 Score	Matthews Correlation Coefficient	Brier Score Loss
Readmitted within 7 days	Logistic Regression	0.101 [0.098-0.106]	0.656 [0.650-0.662]	0.308 [0.206-0.428]	0.003 [0.002-0.005]	0.949 [0.949-0.949]	0.006 [0.004-0.009]	0.027 [0.016-0.040]	0.048 [0.047-0.048]
	Complement Naive Bayes	0.097 [0.093-0.102]	0.640 [0.634-0.648]	0.080 [0.079-0.082]	0.567 [0.555-0.577]	0.647 [0.645-0.653]	0.141 [0.139-0.143]	0.100 [0.096-0.105]	0.247 [0.246-0.247]
	Gaussian Naive Bayes	0.095 [0.092-0.100]	0.637 [0.630-0.644]	0.138 [0.130-0.146]	0.126 [0.100-0.144]	0.915 [0.910-0.923]	0.131 [0.117-0.142]	0.087 [0.079-0.096]	0.074 [0.069-0.078]
	Support Vector Machine	0.104 [0.100-0.108]	0.660 [0.653-0.665]	0.259 [0.072-0.478]	0.001 [0.000-0.001]	0.949 [0.949-0.949]	0.001 [0.000-0.002]	0.010 [0.001-0.020]	0.049 [0.048-0.050]
Readmitted within 3 days	Complement Naive Bayes	0.039 [0.037-0.043]	0.616 [0.607-0.625]	0.033 [0.032-0.033]	0.532 [0.516-0.549]	0.644 [0.641-0.648]	0.061 [0.060-0.063]	0.054 [0.050-0.059]	0.248 [0.247-0.248]
	Logistic Regression	0.041 [0.039-0.044]	0.631 [0.622-0.639]	0.154 [0.000-0.308]	0.001 [0.000-0.002]	0.978 [0.978-0.978]	0.002 [0.000-0.003]	0.009 [-0.002-0.020]	0.021 [0.021-0.021]
	Support Vector Machine	0.042 [0.040-0.045]	0.636 [0.627-0.643]	0.000 [0.000-0.000]	0.000 [0.000-0.000]	0.978 [0.978-0.978]	0.000 [0.000-0.000]	0.000 [0.000-0.000]	0.035 [0.023-0.207]
	Gaussian Naive Bayes	0.037 [0.035-0.039]	0.617 [0.608-0.625]	0.056 [0.051-0.062]	0.091 [0.077-0.123]	0.947 [0.931-0.952]	0.069 [0.063-0.077]	0.045 [0.038-0.053]	0.048 [0.044-0.060]
Readmitted within 30 days	Logistic Regression	0.300 [0.296-0.305]	0.698 [0.694-0.702]	0.541 [0.521-0.565]	0.050 [0.047-0.053]	0.855 [0.855-0.856]	0.091 [0.087-0.097]	0.131 [0.124-0.140]	0.116 [0.115-0.116]
	Complement Naive Bayes	0.290 [0.285-0.294]	0.680 [0.675-0.686]	0.242 [0.240-0.246]	0.615 [0.605-0.620]	0.664 [0.661-0.672]	0.348 [0.344-0.351]	0.210 [0.204-0.214]	0.245 [0.245-0.245]
	Gaussian Naive Bayes	0.287 [0.282-0.291]	0.677 [0.672-0.680]	0.345 [0.333-0.373]	0.249 [0.202-0.269]	0.821 [0.816-0.834]	0.289 [0.262-0.302]	0.193 [0.185-0.201]	0.145 [0.140-0.147]
	Support Vector Machine	0.303 [0.298-0.307]	0.700 [0.696-0.703]	0.591 [0.565-0.618]	0.032 [0.029-0.034]	0.856 [0.855-0.856]	0.060 [0.056-0.065]	0.112 [0.105-0.119]	0.123 [0.119-0.125]
Hospital stay over 7 days	Complement Naive Bayes	0.326 [0.322-0.331]	0.638 [0.634-0.643]	0.395 [0.387-0.403]	0.221 [0.216-0.228]	0.770 [0.769-0.772]	0.284 [0.278-0.291]	0.169 [0.163-0.177]	0.240 [0.240-0.240]
	Gaussian Naive Bayes	0.347 [0.342-0.352]	0.677 [0.674-0.681]	0.404 [0.397-0.411]	0.270 [0.264-0.276]	0.768 [0.766-0.770]	0.323 [0.317-0.330]	0.196 [0.189-0.203]	0.196 [0.194-0.198]
	Support Vector Machine	0.367 [0.361-0.372]	0.693 [0.690-0.697]	0.562 [0.544-0.586]	0.048 [0.045-0.050]	0.797 [0.796-0.798]	0.088 [0.083-0.093]	0.118 [0.110-0.126]	0.168 [0.156-0.179]
	Logistic Regression	0.366 [0.360-0.371]	0.691 [0.688-0.694]	0.551 [0.533-0.568]	0.070 [0.067-0.074]	0.797 [0.796-0.798]	0.124 [0.119-0.130]	0.140 [0.133-0.148]	0.150 [0.150-0.151]
Hospital stay over 5 days	Support Vector Machine	0.530 [0.527-0.532]	0.694 [0.693-0.696]	0.636 [0.632-0.640]	0.221 [0.219-0.224]	0.698 [0.697-0.699]	0.328 [0.325-0.331]	0.233 [0.229-0.235]	0.270 [0.253-0.286]
	Complement Naive Bayes	0.462 [0.460-0.465]	0.610 [0.608-0.612]	0.584 [0.581-0.588]	0.201 [0.198-0.202]	0.686 [0.686-0.687]	0.299 [0.296-0.300]	0.192 [0.190-0.194]	0.240 [0.240-0.240]
	Logistic Regression	0.529 [0.527-0.531]	0.694 [0.693-0.695]	0.625 [0.624-0.629]	0.238 [0.236-0.239]	0.699 [0.698-0.699]	0.344 [0.343-0.346]	0.236 [0.235-0.238]	0.198 [0.198-0.199]
	Gaussian Naive Bayes	0.505 [0.502-0.507]	0.678 [0.677-0.679]	0.581 [0.579-0.584]	0.260 [0.258-0.260]	0.691 [0.690-0.692]	0.359 [0.357-0.360]	0.220 [0.218-0.222]	0.250 [0.248-0.250]
Hospital stay over 3 days	Complement Naive Bayes	0.700 [0.698-0.702]	0.624 [0.622-0.625]	0.741 [0.737-0.743]	0.249 [0.245-0.252]	0.508 [0.506-0.509]	0.372 [0.368-0.376]	0.155 [0.150-0.157]	0.242 [0.241-0.242]

	Gaussian Naive Bayes	0.733 [0.730-0.735]	0.673 [0.671-0.675]	0.760 [0.757-0.764]	0.329 [0.321-0.334]	0.545 [0.541-0.548]	0.459 [0.450-0.464]	0.205 [0.198-0.211]	0.288 [0.285-0.293]
	Logistic Regression	0.753 [0.751-0.755]	0.694 [0.692-0.695]	0.669 [0.668-0.669]	0.820 [0.819-0.821]	0.656 [0.655-0.657]	0.737 [0.736-0.737]	0.266 [0.265-0.268]	0.215 [0.215-0.216]
	Support Vector Machine	0.753 [0.751-0.754]	0.693 [0.691-0.694]	0.667 [0.666-0.667]	0.825 [0.824-0.825]	0.655 [0.655-0.656]	0.737 [0.737-0.738]	0.264 [0.263-0.265]	0.399 [0.369-0.430]
Readmitted within 5 days	Logistic Regression	0.072 [0.069-0.077]	0.646 [0.639-0.653]	0.253 [0.119-0.385]	0.002 [0.001-0.003]	0.963 [0.963-0.963]	0.004 [0.002-0.006]	0.019 [0.007-0.031]	0.035 [0.035-0.035]
	Gaussian Naive Bayes	0.068 [0.065-0.072]	0.629 [0.622-0.637]	0.101 [0.089-0.114]	0.114 [0.091-0.172]	0.929 [0.906-0.938]	0.106 [0.097-0.119]	0.070 [0.062-0.080]	0.062 [0.055-0.080]
	Complement Naive Bayes	0.069 [0.066-0.073]	0.631 [0.621-0.638]	0.057 [0.056-0.058]	0.555 [0.541-0.566]	0.645 [0.642-0.651]	0.104 [0.102-0.106]	0.080 [0.075-0.085]	0.247 [0.247-0.247]
	Support Vector Machine	0.074 [0.070-0.078]	0.651 [0.643-0.657]	0.149 [0.000-1.000]	0.000 [0.000-0.000]	0.963 [0.963-0.963]	0.000 [0.000-0.001]	0.002 [-0.001-0.014]	0.041 [0.036-0.076]

d. Length of Stay predictions without primary diagnosis code, gradient boosting machine

Target	Average Precision	ROC AUC	Precision	Recall	Accuracy	F1 Score	Matthews Correlation Coefficient	Brier Score Loss
Hospital stay over 7 days	0.491 [0.486-0.497]	0.781 [0.778-0.784]	0.635 [0.626-0.647]	0.212 [0.203-0.222]	0.813 [0.812-0.815]	0.317 [0.308-0.328]	0.288 [0.281-0.295]	0.134 [0.133-0.135]
Hospital stay over 5 days	0.640 [0.636-0.644]	0.781 [0.779-0.784]	0.655 [0.650-0.660]	0.439 [0.433-0.445]	0.736 [0.734-0.738]	0.526 [0.520-0.531]	0.367 [0.361-0.372]	0.173 [0.172-0.174]
Hospital stay over 3 days	0.811 [0.808-0.814]	0.762 [0.759-0.765]	0.716 [0.713-0.718]	0.817 [0.814-0.821]	0.702 [0.699-0.705]	0.763 [0.761-0.765]	0.373 [0.367-0.378]	0.192 [0.191-0.193]

Supplementary Table 2. Categorized variables

Outcomes

Length of stay in days
Length of stay over 14 days
Length of stay over 3 days
Length of stay over 5 days
Length of stay over 7 days
Readmitted within 15 days
Readmitted within 180 days
Readmitted within 20 days
Readmitted within 28 days
Readmitted within 3 days
Readmitted within 30 days
Readmitted within 365 days
Readmitted within 3650 days
Readmitted within 45 days
Readmitted within 5 days
Readmitted within 7 days
Readmitted within 90 days

Hospital events and information

Admit source description
Hospital transfer description
Patient class description
Discharge disposition description
Has had at least 1 emergency department encounter in the past 6 months
Number of emergency department visits in past 6 months
Admitted from emergency department
Admitted to intensive care unit at any point during current hospitalization
Admitted to intensive care unit on first day of hospitalization
Discharged from intensive care unit at any point during current hospitalization
Discharged from intensive care unit on first day of hospitalization
Intensive care unit length of stay in days
On dialysis
Received total parenteral nutrition
Pressure ulcer
Pressure ulcer absent
Pressure ulcer information unavailable
Pressure ulcer present on admission to the hospital
Fall during admission
Clostridium difficile infection
Had a line infection during admission
Medications administered in hospital on day of admission
Medications administered in the first 24 hours
Number of prescribed homegoing medications on day of discharge
Opiates administered at any point during admission
Benzodiazepines administered during admission
Discharged with benzodiazepine prescription
Discharged with opiate prescription
Number of medications administered in hospital on day of discharge
Received a physical or occupational therapy consult during admission
Received a palliative care consult during admission
Received a spiritual care consult during admission
Received an infectious disease consult during admission
Received a hospice consult during admission
Number of other patients admitted on same day
Number of other patients discharged on same day
Number of patients having complete blood counts drawn at this time, admission
Number of patients having complete blood counts drawn at this time, discharge
Number of patients having comprehensive metabolic panels drawn at this time, admit
Number of patients having comprehensive metabolic panels drawn at this time, discharge

Patient

Age
Race
Primary language
Marital status

Listed address is likely a new address

Primary diagnosis
Systolic blood pressure on admission
Systolic blood pressure on discharge
Diastolic blood pressure on admission
Diastolic blood pressure on discharge
Temperature on admission
Temperature on discharge
Heart rate on admission
Heart rate on discharge
Body mass index on admission
Body mass index on discharge
Had a hospital discharge in the past 30 days
Length of stay of last admission
Number of past admissions
Medicaid
Medicare
Private health insurance
Insurance other
Insurance provider
Payer class information unavailable

Comorbidities

Anxiety
Cerebral palsy
Cerebrovascular disease
Chronic pulmonary disease
Congestive heart failure
Connective tissue disorder
Dementia
Depression
Diabetes with chronic complication
Diabetes without chronic complication
Epilepsy
Has a diagnosis of AIDS or HIV
Hemiplegia or paraplegia
Hip replacement
Pressure ulcer, history of
Hypertension
Knee replacement
Metastatic solid tumor
Mild liver disease
Moderate or severe liver disease
Myocardial infarction
Number of morbidities
Peptic ulcer disease
Peripheral vascular disease
Pneumonia
Psychosis
Renal disease
Rheumatic disease
Short gut syndrome
Solid organ transplant

Laboratory

Absolute basophil count discharge value
Absolute eosinophil count at discharge
Absolute eosinophil count on admission
Absolute lymphocyte count at discharge
Absolute lymphocyte count on admission

Absolute monocyte count at discharge

Absolute monocyte count on admission

Absolute neutrophil count value on admission

Absolute neutrophil count value on discharge

Absolute nucleated red blood cell count on admission

Absolute nucleated red blood cell count on discharge

Alanine aminotransferase admit value

Alanine aminotransferase discharge value

Albumin admit value

Albumin discharge value

Alkaline phosphatase value at discharge

Alkaline phosphatase value on admission

Anion gap admit value

Anion gap discharge value

Aspartate aminotransferase admit value

Aspartate aminotransferase discharge value

Basophil admit value

Basophil count at discharge

Bilirubin total admit value

Bilirubin total discharge value

Blood urea nitrogen value on admission

Blood urea nitrogen value on discharge

Calcium admit value

Calcium discharge value

Cancer, excluding skin cancers

Carbon dioxide admit value

Carbon dioxide discharge value

Chloride admit value

Chloride discharge value

Creatinine value on admission

Creatinine value on discharge

Eosinophil count admit value

Eosinophil count discharge value

Estimated glomerular filtration rate adjusted for African Americans, admit value

Estimated glomerular filtration rate adjusted for African Americans, discharge value

Estimated glomerular filtration rate adjusted for other races, admit value

Estimated glomerular filtration rate adjusted for other races, discharge value

Glucose value at discharge

Glucose value on admission

Hematocrit value at discharge

Hematocrit value on admission

Hemoglobin value at discharge

Hemoglobin value on admission

Lymphocyte count at discharge

Lymphocyte value on admission

Mean corpuscular hemoglobin concentration admit value

Mean corpuscular hemoglobin concentration value on discharge

Mean corpuscular hemoglobin value at discharge

Mean corpuscular hemoglobin value on admission

Mean corpuscular volume value on admission

Mean corpuscular volume value on discharge

Mean platelet volume admit value

Mean platelet volume discharge value

Monocyte count admit value

Monocyte count discharge value

Neutrophil count at discharge

Neutrophil value on admission

Nucleated red cell count admit value

Nucleated red cell count discharge value

Platelet count at discharge

Platelet count on admission

Potassium admit value

Potassium discharge value

Protein total admit value

Protein total discharge value

Red blood cell count at discharge

Red blood cell value on admission

Red cell distribution width value on admission

Red cell distribution width value on discharge

Sodium admit value

Sodium discharge value

White blood cell count on discharge

White blood cell value on admission

<u>Timing</u>

Admission date, numeric week of year

Admission day of week

Admitted between 10pm and 12am

Admitted between 12am and 4am

Admitted between 4am and 7am

Admitted between 6pm and 10pm

Admitted between 7am and 6pm

Admitted on holiday

Admitted on weekend

Comprehensive metabolic panel admit time during working day 7am 6pm

Comprehensive metabolic panel admit time early morning 4am 7am

Comprehensive metabolic panel admit time hour of day

Comprehensive metabolic panel admit time month

Comprehensive metabolic panel admit time on holiday

Comprehensive metabolic panel admit time on weekend

Comprehensive metabolic panel admit time quarter

Comprehensive metabolic panel discharge time at night 6pm 10pm

Comprehensive metabolic panel discharge time day of week

Comprehensive metabolic panel discharge time during working day 7am 6pm

Comprehensive metabolic panel discharge time early morning 4am 7am

Comprehensive metabolic panel discharge time hour of day

Comprehensive metabolic panel discharge time month

Comprehensive metabolic panel discharge time on holiday

Comprehensive metabolic panel discharge time on weekend

Day of week on discharge

Days between current admission and previous discharge

Discharge between 4am and 7am

Discharge date, numeric week of year

Discharge hour of day

Discharged between 10pm and 12am

Discharged between 12am and 4am

Discharged between 6pm and 10pm

Discharged between 7am and 6pm

Discharged on holiday

Discharged on weekend

Discharged quarter of year

First complete blood count drawn on weekend

First complete blood count resulted between 12 and 4am

First complete blood count resulted between 4am and 7am

First complete blood count resulted between 6pm and 10pm

First complete blood count resulted between 7am and 6pm

First complete blood count resulted day of week
First complete blood count resulted on a US holiday
First comprehensive metabolic panel resulted between 10pm and 12am
First comprehensive metabolic panel resulted between 12am and 4am
First comprehensive metabolic panel resulted between 6pm and 10pm
First comprehensive metabolic panel resulted day of week
Last complete blood count resulted between 10pm and 12am
Last complete blood count resulted between 10pm and 12am
Last complete blood count resulted between 12am and 4am
Last complete blood count resulted between 4 and 7am
Last complete blood count resulted between 6 and 10pm
Last complete blood count resulted between 7am and 6pm
Last complete blood count resulted on a US holiday
Last complete blood count resulted on a weekend day
Last comprehensive metabolic panel resulted between 10pm and 12am
Last comprehensive metabolic panel resulted between 12am and 4am
Month of the year when first complete blood count resulted
Month of year at discharge
Numeric hour of day of admission
Numeric hour of day when first complete blood count resulted
Numeric hour of day when last complete blood count resulted
Numeric month of year on admission
Numeric month of year when last complete blood count resulted
Numeric quarter of the year when first complete blood count resulted
Numeric quarter of year on admission
Numeric quarter of year when last complete blood count resulted
Numeric quarter of year when last comprehensive metabolic panel resulted
Numeric week of the year, last complete blood count resulted
Numeric week of year
Numeric week of year, first comprehensive metabolic panel resulted
Numeric week of year, last comprehensive metabolic panel resulted
Weekday of last complete blood count result

<u>American Community Survey (Census Information)</u>

American Community Survey: Average size of households, renters and owners
American Community Survey: BlockGroup GeoID
American Community Survey: Employed population count
American Community Survey: Income below 50 percent of poverty level
American Community Survey: Income between 100 and 124 percent of poverty level
American Community Survey: Income between 125 and 149 percent of poverty level
American Community Survey: Income between 150 and 184 percent of poverty level
American Community Survey: Income between 185 and 199 percent of the poverty ratio
American Community Survey: Income between 50 and 99 percent of poverty level
American Community Survey: Income over twice the poverty level
American Community Survey: Median age
American Community Survey: Median age of females
American Community Survey: Median age of males
American Community Survey: Number of families with female householder, no husband present
American Community Survey: Number of families with male householder, no wife present
American Community Survey: Number of householders living alone
American Community Survey: Number of householders not living alone
American Community Survey: Number of married couple families
American Community Survey: Population employed
American Community Survey: Population identifying as two or more races
American Community Survey: Population unemployed
American Community Survey: Race, black alone
American Community Survey: Race, white alone
American Community Survey: Total population count
American Community Survey: Total population not in labor force

References

1. Milinovich A, Kattan MW. Extracting and utilizing electronic health data from Epic for research. Ann Transl Med 2018;6(3).

2. Lind KD, Noel-Miller CM, Sangaralingham LR, et al. Increasing Trends in the Use of Hospital Observation Services for Older Medicare Advantage and Privately Insured Patients. Med Care Res Rev 2019;76(2):229–39.

3. Zuckerman RB, Sheingold SH, Orav EJ, Ruhter J, Epstein AM. Readmissions, Observation, and the Hospital Readmissions Reduction Program. N Engl J Med 2016;374(16):1543–51.

4. Natekin A, Knoll A. Gradient boosting machines, a tutorial. Front Neurorobotics 2013;7:21.

5. Ke G, Meng Q, Finley T, et al. LightGBM: A Highly Efficient Gradient Boosting Decision Tree [Internet]. In: Guyon I, Luxburg UV, Bengio S, et al., editors. Advances in Neural Information Processing Systems 30. Curran Associates, Inc.; 2017. p. 3146–3154.Available from: http://papers.nips.cc/paper/6907-lightgbm-a-highly-efficient-gradient-boosting-decision-tree.pdf

6. Lundberg SM, Erion G, Chen H, et al. Explainable AI for Trees: From Local Explanations to Global Understanding. 2019.

7. Lundberg SM, Lee S-I. A Unified Approach to Interpreting Model Predictions [Internet]. In: Guyon I, Luxburg UV, Bengio S, et al., editors. Advances in Neural Information Processing Systems 30. Curran Associates, Inc.; 2017. p. 4765–4774.Available from: http://papers.nips.cc/paper/7062-a-unified-approach-to-interpreting-model-predictions.pdf

8. Steyerberg EW, Vickers AJ, Cook NR, et al. Assessing the performance of prediction models: a framework for traditional and novel measures. Epidemiol Camb Mass 2010;21(1):128–138.

9. Pencina MJ, D'Agostino RB. Evaluating discrimination of risk prediction models: the C statistic. Jama 2015;314(10):1063–1064.

10. Chai T, Draxler RR. Root mean square error (RMSE) or mean absolute error (MAE)?–Arguments against avoiding RMSE in the literature. Geosci Model Dev 2014;7(3):1247–1250.
